# Diverse ssRNA viruses associated with *Karenia brevis* harmful algal blooms in southwest Florida

**DOI:** 10.1128/msphere.01090-24

**Published:** 2025-03-20

**Authors:** Shen Jean Lim, Alexandra Rogers, Karyna Rosario, Makenzie Kerr, Matt Garrett, Julie Koester, Katherine Hubbard, Mya Breitbart

**Affiliations:** 1 College of Marine Science, University of South Florida, St. Petersburg, Florida, USA; 2 Florida Fish and Wildlife Conservation Commission-Fish and Wildlife Research Institute, St. Petersburg, Florida, USA; Third Institute of Oceanography Ministry of Natural Resources, Xiamen, China

**Keywords:** harmful algal bloom, HAB, red tide, *Karenia brevis*, virus, RNA virus, Florida, Gulf of Mexico

## Abstract

Harmful algal blooms (HABs) caused by the dinoflagellate *Karenia brevis* frequently occur in the eastern Gulf of Mexico, where they negatively impact the environment, human health, and economy. Very little is known about viruses associated with *K. brevis* blooms, although viral infection of other HAB-forming phytoplankton species can play an important role in bloom dynamics. We used viral metagenomics to identify viruses in 11 pooled seawater samples collected from southwest Florida, USA, in 2021 during a severe, spatiotemporally dynamic *K. brevis* bloom. Assembled viral genomes were similar to published genomes from the order *Picornavirales*, family *Marnaviridae*, and genera *Sogarnavirus*, *Bacillarnavirus*, and *Marnavirus*. Several of the cultured viruses from these groups infect bloom-forming diatoms (*Chaetoceros* sp. and *Rhizosolenia setigera*) and the raphidophyte *Heterosigma akashiwo*. We also recovered unclassified *Riboviria* genomes related to a *Symbiodinium* positive-sense ssRNA virus sequenced from coral dinoflagellate symbionts. Reverse-transcriptase PCR assays were performed to monitor the occurrence of seven representative virus genomes in these samples from 2021 and 43 seawater samples collected during a subsequent, typical bloom between November 2022 and May 2023. Over half of the samples contained multiple viruses, and at least one viral genome was detected in 44 of the 54 samples collected across seasons and years, highlighting the ubiquity of these viruses in this region. Alpha diversity was highest in the summer months and positively correlated with *K. brevis* cell counts. Multiple regression revealed month and the presence of unclassified *Riboviria* sequences most similar to dinoflagellate viruses as significant predictors of *K. brevis* cellular abundance.

**IMPORTANCE:**

Harmful algal blooms caused by the dinoflagellate *Karenia brevis* negatively impact the tourism, fisheries, and public health sectors. Anticipated impacts of climate change, nutrient pollution, and ocean acidification may sustain and/or exacerbate *K. brevis* blooms in the future, underscoring the need for proactive monitoring, communication, and mitigation strategies. This study represents a pioneering effort in monitoring viruses associated with *K. brevis* blooms. The findings lay the groundwork for studying the effects of environmental drivers on *K. brevis* blooms and their associated viruses, as well as for exploring the roles of viruses in bloom dynamics and potential applications of viruses as biocontrol agents for *K. brevis* blooms. Furthermore, the comparison of viral dynamics relative to local and regional bloom dynamics in this study helps inform future monitoring and modeling needs.

## INTRODUCTION

The dinoflagellate species *Karenia brevis* forms harmful algal blooms (HABs), commonly known as “red tides”, almost annually along the coasts and offshore waters of the eastern Gulf of Mexico (1, 2). Extensive *K. brevis* blooms cause water discoloration and, in some cases, hypoxia (3). *K. brevis* also produces brevetoxins that exhibit neurotoxic (4) or hemolytic (5) activity. These brevetoxins are ichthyotoxic and cause fish kills upon direct exposure (6). Brevetoxins and their metabolites can also accumulate in filter-feeding zooplankton and shellfish, where they are transferred up the food chain (7, 8). This trophic transfer contributes to fish, bird, and marine mammal mortalities, as well as neurotoxic shellfish poisoning (NSP) in humans (9). *K. brevis* cells are readily lysed by high turbulence at the water surface or along the shore, releasing brevetoxins into the water (10). Released brevetoxins can be further aerosolized and transported onshore by winds, where they trigger respiratory irritation in animals (11, 12) and humans (9, 13). The negative aesthetic, environmental, and health effects of *K. brevis* blooms lead to significant economic losses across the tourism, fisheries, and public health sectors (14). These impacts are evident in the Gulf of Mexico, particularly on the west coast of Florida, which experiences the most frequent and persistent occurrences of *K. brevis* blooms (2).

Florida prioritizes strategies that aim to reduce the ecological, health, and economic consequences of *K. brevis* blooms (15). These include collaborative efforts for monitoring and forecasting blooms and related impacts, such as the FWC Historical HAB Database, which compiles and hosts *K. brevis* cell abundance data from 1953 to the present (https://myfwc.com/research/redtide/monitoring/database/), specialized remote-sensing techniques (16), and ocean circulation models (17). These data are integrated to inform shellfish harvesting management (18), forecasts published by NOAA’s National Centers for Coastal Ocean Science (https://coastalscience.noaa.gov/science-areas/habs/hab-forecasts/gulf-of-mexico/) and the University of South Florida (19), and related public outreach initiatives. Biological, chemical, and physical factors are thought to underlie variability in bloom severity and duration (6, 20, 21). However, accurately modeling complex bloom dynamics has proven challenging and is an ongoing process (22). It is critical to identify and/or parameterize ecological metrics and strategies that are relevant for growth and loss at varying scales. This is helpful for building complexity into forecasting models and hindcasting past events.

*K. brevis* blooms can endure for a few months to a few years (23) and can be localized to a single estuary, or span Florida’s Gulf and Atlantic coasts (2). A *K. brevis* bloom event can be broken down into stages, including initiation, growth, maintenance, decline, and termination (21). These stages can occur regionally or locally; for example, a single bloom may initiate only once but may pass through a certain area multiple times during a bloom event. In addition to physical and chemical influences, biotic mechanisms are thought to play important roles in various *K. brevis* bloom stages. These include interactions between *K. brevis* and grazers, parasites, other algal species, algicidal bacteria, and lytic viruses, as well as responses of *K. brevis* to other internal and external triggers, such as life history stage and photosynthesis (6, 20, 21). Understanding biological interactions between HAB species and microbes is crucial, as these interactions can critically influence bloom dynamics and biogeochemical cycling. Furthermore, climate change, with its cascading effects on water temperatures, ocean stratification, currents, and nutrient transport, is predicted to alter HAB interactions, dispersal, and frequency (24).

Virus-host interactions can shape HAB life cycles and serve important roles in bloom ecology (24). Factors such as viral lysis can result in bloom decline and may provide useful insights into bloom prevention and treatment approaches (2, 15). Although both DNA and RNA viruses can infect HAB species (25), most research has focused on single-stranded RNA (ssRNA) viruses, which are prevalent in HAB-forming dinoflagellate, diatom, and raphidophyte species (26). To date, viruses associated with *K. brevis* blooms have not yet been sequenced. An unknown microorganism with lytic activity against *K. brevis* cultures was previously recovered from surface waters containing *K. brevis* blooms in southwest Florida (27). These *K. brevis* cultures contained virus-like particles (VLPs) in their supernatants. However, lysing cells contained mostly bacteria and rarely VLPs, suggesting that *K. brevis* cell death may be caused by virus-bacteria interactions.

In this study, we aimed to identify and monitor RNA viruses in seawater from southwest Florida associated with *K. brevis* blooms. Using viral metagenomics, we recovered near-complete viral genomes from surface water samples collected from 11 locations around Pinellas and Manatee counties in April, June, and July 2021, during a long-lasting and severe *K. brevis* bloom event that began the prior year. We then designed reverse-transcriptase PCR (RT-PCR) primers for each representative viral genome to analyze their presence and ecology in the samples from 2021 and 43 additional seawater samples collected from 39 locations around southwest Florida during a subsequent *K. brevis* bloom event occurring between November 2022 and May 2023. Blooms of *K. brevis* typically start during fall and terminate in or before spring, and as in 2021, occasionally persist through summer. Herein, we use the term bloom event to describe the bloom from initiation through termination, in recognition that blooms typically span multiple seasons and years.

## MATERIALS AND METHODS

### Virome preparation

**Fig 1:**
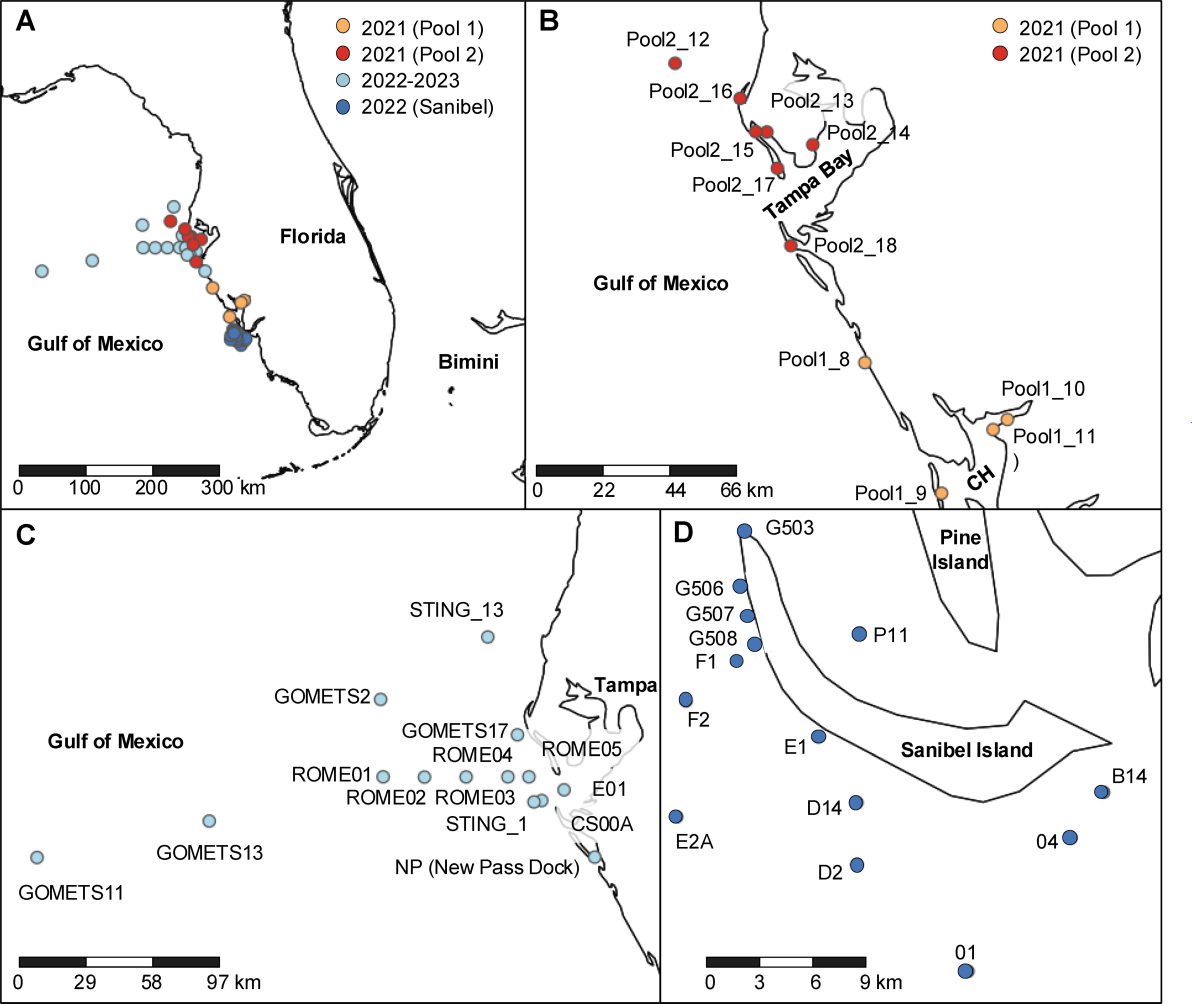
Map of locations of (**A**) all sampling sites, including (**B**) sites from the greater Charlotte Harbor (CH) and Tampa Bay regions where Pool 1 and Pool 2 samples were collected for virome sequencing in 2021; (**C**) other sites in, around, and offshore of Tampa Bay where samples were collected from 2022 to 2023; and (**D**) Sanibel Island sites where samples were collected in 2022. Seawater samples from all sites were collected for RT-PCR and their associated metadata are described in Table 1 and Table S1.

Seawater samples for virome sequencing were collected from four sites in April 2021, three sites in June 2021, and four sites in July 2021 (Fig. 1B; Table S1). From each site, 500 mL of surface water was collected in an acid-washed or autoclaved bottle and transported to the laboratory on ice. In the lab, each water sample was filtered through a 50 mm 0.45 µm Nalgene Rapid-Flow Sterile Disposable Filter Unit with PES membrane (Thermo Scientific, Waltham, MA, USA). Each filter was placed in a sterile disposable petri dish and cut in half with a sterile razor blade. Half of the filter was homogenized for virome sequencing, whereas the other half was left in the petri dish, which was wrapped with aluminum foil and parafilm and stored at −80°C for RT-PCR. To ensure complete homogenization, half of the filter used for virome sequencing was cut evenly into four sections. Each section was homogenized in a separate Zymo Research (ZR) BashingBead Lysis Tube (Irvine, CA, USA) containing 2 mm ceramic beads and 1 mL suspension medium (100 mM NaCl, 50 mM Tris-HCl (pH = 7.5), and 8 mM MgSO_4_) for 1.5 min at maximum speed (5 meters/s) using a Fisherbrand Bead Mill 4 Homogenizer (Fisher Scientific, Waltham, MA, USA). Each resulting homogenate was briefly centrifuged, and 950 µL aliquots from each of the four homogenates per sample were pooled for the purification of virus-like particles (VLPs). Each pooled homogenate was centrifuged at 10,000 *g* for 10 min at 4°C, and the supernatant was syringe-filtered through a 0.45 µm Sterivex filter (MilliporeSigma, Burlington, MA, USA). Half of the filtrate was treated with a nuclease mixture of 21 U of TURBO DNase (Invitrogen, Waltham, MA, USA), 4.5 U of Baseline-ZERO DNase (Epicentre, Paris, France), 112.5 U Benzonase endonuclease (MilliporeSigma), and 400 U Ambion RNase I in 1× Turbo DNase Buffer (Invitrogen) for 1.5 h at 37°C, followed by nuclease inactivation with 20 mM EDTA (pH = 8.0) for identification of RNA viruses. The other half of the filtrate was treated similarly but without RNase I for future identification of DNA viruses.

NGS library preparation was performed using methods described in Rosario et al. (28). Briefly, 50 µL of RNA was extracted from each purified RNA virus fraction using Qiagen’s RNeasy Mini kit (Valencia, CA, USA) with on-column DNase digestion and quantified using the Qubit RNA high sensitivity (HS) assay. Two RNA pools from seawater samples collected in April 2021 and from June to July 2021 (Table S1) were initially processed for sequencing. Subsequently, seven individual RNA samples from Pool2 (June–July 2021) were processed to improve the coverage of viral sequences for genome assembly. From each sample, cDNA was synthesized from 8 µL RNA and 50 ng random hexamers using the SuperScript IV First-Strand Synthesis System (Invitrogen). Second-strand synthesis was performed on each cDNA product using New England Biolabs’ DNA Polymerase I, Large (Klenow) Fragment (Ipswich, MA, USA). Double-stranded cDNA samples were purified with ZR’s DNA Clean & Concentrator-25 kit and fragmented to 300 bp at the University of South Florida’s Genomics Program Sequencing core (Tampa, FL, USA) using the Covaris M220 Focused-ultrasonicator (Woburn, MA, USA). NGS libraries were prepared from fragmented cDNA samples using the xGen ssDNA & Low-Input DNA Library Preparation Kit (Integrated DNA Technologies, Coralville, IA, USA) following the manufacturer’s protocol for DNA concentrations < 1 ng/µL. All libraries were paired-end sequenced (2 × 150 bp) by Azenta Life Sciences (Burlington, MA, USA) on an Illumina HiSeq platform.

### Virome assembly and sequence analysis

Sequencing reads were trimmed using the default parameters of Trimmomatic v0.39 (29) with a custom read head crop of 10 bases, as described in Rosario et al. (28). Read quality pre- and post-trimming, including the presence of adapter sequences, was evaluated using FastQC v0.11.8 (https://www.bioinformatics.babraham.ac.uk/projects/fastqc). To obtain near-complete or complete draft viral genomes, several assembly approaches were used. Trimmed reads from each pooled cDNA library (April 2021 and June–July 2021) were initially assembled using the pipeline recommended for PCR-amplified viral metagenomes (30) which included read deduplication using BBtool’s clumpify.sh (dedupe subs = 0 passes = 2; https://sourceforge.net/projects/bbmap), followed by assembly using single-cell SPAdes (31). Trimmed reads from each individual and pooled cDNA library were also individually assembled using the default options of the newer rnaviralSPAdes assembler specific for RNA viruses (32) integrated in SPAdes v3.15.5 (31). Additionally, reads from all sequenced libraries were also combined, deduplicated using BBtool, and co-assembled using rnaviralSPAdes (32).

RNA viral contigs were identified from each assembly using VirBot (33). Sequences of RNA viral contigs ≥ 1,000 nt were compared with sequences from NCBI’s nucleotide (nt) and non-redundant (nr) protein collections (34) using the megablast and/or blastx programs on NCBI’s Basic Local Alignment Research Tool (BLAST) server (35) to identify genome relatives. Contigs homologous to RNA viruses associated with eukaryotic phytoplankton were assessed for quality and completeness using the default parameters of CheckV v1.0.1 (36). Draft genomes with ≥80% completeness (Table 1) were retained for assembly correction, genome annotation, and primer design. For assembly correction, trimmed reads from each library were first mapped to each contig using the default options of bowtie2 v2.2.5 (37). Each resulting SAM (Sequence Alignment Map) file was converted to a sorted and indexed BAM (Binary Alignment Map) using samtools v1.9 (38). The sequence and BAM alignments of each contig were provided as input to Pilon v1.24 (39) to automatically fix nucleotide differences, insertions/deletions, gaps, and local assemblies in the draft genome, using the default parameters. For each base in the input genome, Pilon (39) evaluates whether the base call is supported by a majority of evidence from read alignments, which are weighted by base and mapping quality. Open reading frames (ORFs) in each corrected genome were identified using NCBI’s Open Reading Frame Finder (ORF Finder) web tool (https://www.ncbi.nlm.nih.gov/orffinder). For gene/protein annotation, genome sequences were compared against annotated RNA helicase, RNA-dependent RNA polymerase (RdRp), and viral protein (VP) sequences in related genomes using the tblastn program on NCBI’s BLAST server (35). To annotate the conserved structural protein domains VP1-VP3, reference protein sequences from *Heterosigma akashiwo* RNA virus (HaRNAV) were used (40). Genome organization was visualized using the ggplot2 v3.5.1 (41) R package with the gggenes v0.5.1 extension (https://doi.org/10.32614/CRAN.package.gggenes).

**TABLE 1:** Features of ssRNA viral genomes assembled in this study^*a*^

Genome ID	NCBI accession	Library	Assembly method	Genome length (nt)	Genome completeness (%)	Taxonomy	Host species (type)
Riboviria1_1*	PP297088	All pooled	Co-assembly (rnaviralSPAdes)	6,078	100	*Viruses; Riboviria*	Unknown (dinoflagellate)
Riboviria1_2	PP297086	Pool2	rnaviralSPAdes	5,901	100		
Riboviria1_3	PP297087	Pool2	Single-cell SPAdes	6,058	100		
Riboviria_2*	PP297085	All pooled	Co-assembly (rnaviralSPAdes)	4,158	91.30	*Viruses; Riboviria*	Unknown (dinoflagellate)
Sogarnavirus1_1*	PP297083	Pool2	Single-cell SPAdes	9,790	100	*Viruses; Riboviria; Orthornavirae; Pisuviricota; Pisoniviricetes; Picornavirales; Marnaviridae; Sogarnavirus*	Unknown (diatom)
Sogarnavirus1_2	PP297084	All pooled	Co-assembly (rnaviralSPAdes	7,225	80.35		
Sogarnavirus2_1*	PP297079	All pooled	Co-assembly (rnaviralSPAdes)	9,652	100	*Viruses; Riboviria; Orthornavirae; Pisuviricota; Pisoniviricetes; Picornavirales; Marnaviridae; Sogarnavirus; Chaetarnavirus 2*	*Chaetoceros* sp. (diatom)
Sogarnavirus2_2	PP297078	Pool2_14	rnaviralSPAdes	9,574	100		
Sogarnavirus2_3	PP297080	Pool2	rnaviralSPAdes	9,575	100		
Sogarnavirus3*	PQ118637	All pooled	Co-assembly (rnaviralSPAdes)	8,488	92.96	*Viruses; Riboviria; Orthornavirae; Pisuviricota; Pisoniviricetes; Picornavirales; Marnaviridae; Sogarnavirus; Chaetarnavirus 2*	*Chaetoceros* sp. (diatom)
Bacillarnavirus1*	PP297082	All pooled	Co-assembly (rnaviralSPAdes)	8,983	96.82	*Viruses; Riboviria; Orthornavirae; Pisuviricota; Pisoniviricetes; Picornavirales; Marnaviridae; Bacillarnavirus;* Rhizosolenia setigera RNA virus 01	*Rhizosolenia setigera* (diatom)
Marnavirus1*	PP297081	All pooled	Co-assembly (rnaviralSPAdes)	7,710	88.66	*Viruses; Riboviria; Orthornavirae; Pisuviricota; Pisoniviricetes; Picornavirales; Marnaviridae; Marnavirus*	Unknown (raphidophyte)

^*a*^ Representative genomes for each viral taxon are indicated with asterisks in the first column.

Nucleotide sequences of all newly assembled genomes were aligned with sequences of their genome relatives that were identified by BLAST using the L-INS-I and --adjustdirectionaccurately options on the MAFFT v7 server (42). Amino acid sequences of the RdRp and capsid protein were also extracted from the annotation data of each viral genome for multiple sequence alignment (MSA). The MSA template used for RdRp sequences was a previously published MSA (43), spanning eight conserved domains (44), that was used by the International Committee on Taxonomy of Viruses (ICTV) for *Marnaviridae* genus/species demarcation (45). This RdRp reference MSA was reconstructed using the MAFFT v7 server (42), followed by alignment editing using Unipro UGENE v48.1 (46). The MSA template used for capsid protein sequences was downloaded from the Pfam database (47) (Pfam entry: PF08762). This protein family was identified using the InterProScan online tool (48), using capsid protein sequences assembled in this study as input. The Pfam capsid protein MSA, which contained 204 sequences, was pruned to retain 83 sequences with stable phylogenetic positions that are consistent with the phylogeny reported in (43) and in the ICTV Report on *Marnaviridae* (45). RdRp and capsid protein sequences assembled in this study were added to their respective reference alignments using the --add, --keeplength, and L-INS-I options on the MAFFT v7 server (42). Pairwise sequence identities were computed from each MSA, with alignment gaps included, using the “Similarity” algorithm of the “Generate distance matrix” function in Unipro UGENE v48.1 (46). Phylogenetic analysis was performed on the RdRp (118 aa) and capsid protein (746 aa) MSA according to the methods described in (43). PhyML 3.0 (49), as implemented on the NGPhylogeny.fr server (50), was used to generate a maximum likelihood tree from each MSA using the default LG + I + G + F amino acid model and default random seed of 123456. For tree topology search, the best of NNI (Nearest Neighbor Interchange) or SPR (Subtree Pruning and Regraphing) was selected. Branch support was determined using the Shimodaira-Hasegawa (SH)-like statistical test (51). All trees were annotated using FigTree v1.4.4 (http://tree.bio.ed.ac.uk/software/Figtree).

For read coverage analysis, the longest genome of each viral taxon (Table 1) was selected as the representative genome and used to build an index using bowtie2 v2.5.0 (37) and a bin collection in anvi’o v8 (52). Trimmed reads from each library were mapped to the Bowtie2 index and converted into a sorted and indexed BAM file using bowtie2 (37) and samtools v1.16.1 (38). All generated BAM files were imported into anvi’o (52) using the anvi-profile command and merged into a single profile using the anvi-merge command. The anvi-summarize command was used to output mean coverage and mean variability (number of reported single nucleotide variants per kilobase pair; SNVs/Kb) values of each representative genome from the merged profile. To minimize non-specific mapping, the mean Q2Q3 coverage, calculated as the average depth of coverage for nucleotide positions that fall between the second and third quartiles of the coverage distribution for each genome, was used. For mean variability calculations, only positions with coverage values ≥ 10 were used. The anvi-gen-variability-profile command was used to analyze the variability of single nucleotide positions, measured as Shannon entropy (53), in each representative genome. To further reduce non-specific mapping, only positions with non-outlier coverage values compared to all other positions in each genome (cov_outlier_in_contig = 0) were included.

### Primer design

RT-PCR primers were designed to amplify conserved genes in the representative genomes of each viral taxon using the modified Primer3 software implemented in the Geneious Prime 2023 full release (54) or the PrimerQuest tool in the Integrated DNA Technologies (IDT, Skokie, IL, USA) web server, with the following parameters: Tm: 58-60°C, GC content: 50%–60%, and primer size: 15–30 bp. Candidate primer sequences (Table S2) were evaluated for genome specificity via sequence searches using Unipro UGENE v48.1 (46) against all genomes assembled in this study, which include target and non-target genomes, as well as sequence searches against all organisms in NCBI’s nr nucleotide collection (34) using the default parameters of Primer-BLAST (55). Newly designed primer pairs were tested and optimized on pooled cDNA from the 11 seawater samples used for virome sequencing. A gradient PCR reaction was performed to determine the optimal annealing temperature of each primer pair. The reaction contained 0.48 µM of each primer, 2 µL cDNA template, 1 µL GC enhancer, and 1× AmpliTaq Gold 360 Master Mix (Applied Biosystems, Waltham, MA, USA) in a 25 µL reaction volume. Gradient PCR was performed under the following conditions: Initial denaturation at 95°C for 10 min, 40 cycles of denaturation at 95°C for 30 s, annealing at 53-59.9°C for 30 s, extension at 72°C for 1 min, followed by elongation at 72°C for 10 min and cooling at 11°C. PCR products were visualized following gel electrophoresis on a 2% (wt/vol) agarose gel stained with ethidium bromide. For each primer set, the annealing temperature that produced the brightest band with no nonspecific amplification was chosen for subsequent RT-PCR assays. PCR products were purified using the Zymoclean Gel DNA Recovery Kit, quantified using the Qubit DNA high sensitivity (HS) assay (Invitrogen), and Sanger sequenced bidirectionally by TACGen (Richmond, CA, USA) or Eurofin Genomics (Louisville, KY, USA).

### RT-PCR

RT-PCR assays using each newly designed primer pair were performed on the 11 samples used for virome sequencing (Table S3), 43 additional seawater samples collected during a subsequent *K. brevis* bloom event (Table S1), and 62 samples from cell cultures of *Karenia* spp. (Table S4). The 43 seawater samples were collected by the Fish and Wildlife Research Institute (FWRI) from around southwest Florida between 2022 and 2023. The volume of collected seawater samples ranged from 500 mL to 2 L, and each sample was filtered through a 50 mm, 0.45 µm filter as described above. The 62 cell culture samples were obtained from 17 strains of *Karenia* spp. grown in three independent flasks at FWRI; two replicates were sampled in the mid-exponential phase, and the third was harvested in the stationary phase. *K. brevis* strains CCFWC 242, 254, 257, 258 (only two replicates available), 261, 267, 121, 123, 124, 125, 126, 1010, 1012, 1013, 1014, 1016, and 1021, *Karenia mikimotoi* strain CCFWC 67, *Karenia papilionacea* strain CCFWC 1020, *Karenia umbella* strain 1019 B2, and media blanks were individually filtered through 25 mm, 0.45 µm Durapore PVDF membrane filters (MilliporeSigma). The volume of culture filtered ranged from 9 mL to 33 mL. From each filtered field-collected or culture sample, a quarter of each filter was cut out, homogenized, and used for total RNA extraction, quantification, cDNA synthesis, and RT-PCR using the methods described above.

### Viral diversity analysis

Location data for field-collected samples were used to create maps with SimpleMappr (https://www.simplemappr.net). *K. brevis* counts were downloaded from FWRI’s harmful algal bloom (HAB) database at https://geodata.myfwc.com/datasets/myfwc::recent-harmful-algal-bloom-hab-events (Table S5). Cell counts for each field-collected sample were matched to the exact or closest sample collection date, depth, latitude, and longitude. RT-PCR results from all field-collected samples were combined to generate a presence/absence matrix for diversity analysis, where presence = 1 and absence = 0. The RT-PCR data matrix and sample metadata were separately imported into R v4.0.2. Diversity analysis was performed using the ampvis2 v2.7.34 R package (56), which converted the RT-PCR data into an Operational Taxonomic Unit (OTU) table, where each OTU indicates a representative genome from each viral taxon (Table 1). This OTU table was linked to the sample metadata and stored as an ampvis2 object. Alpha diversity was computed with the amp_alphadiv function using the observed OTU measure. Shapiro-Wilk tests, implemented in R, rejected the null hypothesis (*P* < 0.05) that alpha diversity and numerical environmental variables (latitude, longitude, depth, and *K. brevis* cell counts) were normally distributed. Therefore, non-parametric statistical tests were used for downstream analyses with a statistical significance threshold of *P* < 0.05. Alpha diversity between sample groups, clustered by year, season, month, or *K. brevis* cell count category, was compared using Kruskal-Wallis rank sum tests (57) implemented in R. *K. brevis* cell count category was defined according to FWRI’s criteria (https://www.flickr.com/photos/myfwc/sets/72157635398013168) of not present/background (0–1,000 *K. brevis* cells/L); very low (>1,000–10,000 *K*. *brevis* cells/L); low (>10,000–100,000 *K*. *brevis* cells/L); medium (>100,000–1,000,000 *K*. *brevis* cells/L); and high (>1,000,000 *K*. *brevis* cells/L). Correlations between alpha diversity and numerical environmental variables were evaluated using the Kendall rank correlation test implemented in the cor.test function in R. Kendall rank correlation was recommended for small, non-parametric data sets with many repeated ranks (58). Alpha-diversity results were visualized using the ggplot2 v3.5.1 (41) R package with the ggbeeswarm v0.7.2 extension (https://doi.org/10.32614/CRAN.package.ggbeeswarm). Beta-diversity was computed and visualized with ampvis2’s amp_ordinate function using the principal components analysis (PCA) method with presence/absence transformation. Environmental factors were fitted onto the resulting PCA plot using the envfit function in the VEGAN v2.6.4 R package (59), which reports a goodness of fit R^2^ value and *P*-value for each variable.

### Multiple regression

Multiple regression models were fitted to analyze associations between predictor variables (environmental factors) and the response variable alpha diversity, as well as predictor variables (environmental factors and virus presence/absence) and the response variable *K. brevis* cell counts. All models were initially created using the lmp function of the lmperm v2.1.0 R package (https://doi.org/10.32614/CRAN.package.lmPerm), which uses permutation tests instead of the normal distribution to calculate *P*-values. Each model was optimized with a stepwise algorithm that adds and removes predictor variables from the model until it reaches the lowest possible Aikake information criterion (AIC) value (60), using the stepAIC function of the Modern Applied Statistics with S (MASS v7.3.60) R package (61). Multicollinearity between predictor variables in each optimized model was detected using a generalized variance inflation factor (GVIF) threshold of ≥4, which corresponds to GVIF ^(1/(2xdf))^ ≥2. GVIF values were calculated using the vif function in the MASS package (61), with the type argument set to “predictor.” Highly correlated variables, if any, were removed incrementally from the initial model, and the model was re-optimized with stepAIC. This process was repeated until a final model with no multicollinearity was generated. Statistically significant predictors in each final model were identified using analysis of variance (ANOVA) tests implemented in R.

## RESULTS

### Viral genomes

Sampling occurred during an unusually long bloom event from December 2020 to November 2021 (62), and at the time of year when most blooms have subsided (FWC Historical HAB Database). We sequenced the viromes of 11 surface seawater samples collected in Pinellas and Manatee counties in April (*n* = 4), June (*n* = 3), and July 2021 (*n* = 4) (Fig. 1B; Table S1). Two pooled libraries comprising samples collected from the greater Charlotte Harbor region in April 2021 (Pool 1) and the greater Tampa Bay region in June-July 2021 (Pool 2) were initially sequenced and analyzed. Sequences related to ssRNA viruses infecting eukaryotic phytoplankton were only detected in Pool 2. To improve the coverage of viral sequences for genome assembly, viromes from each of the seven samples in Pool 2 were also individually sequenced (Fig. 1B; Table S1). From these pooled and individually sequenced libraries, we assembled 12 viral genomes (Table 1) with ≥80% completeness using single-cell SPAdes (31) or rnaviralSPAdes (32).

**Fig 2:**
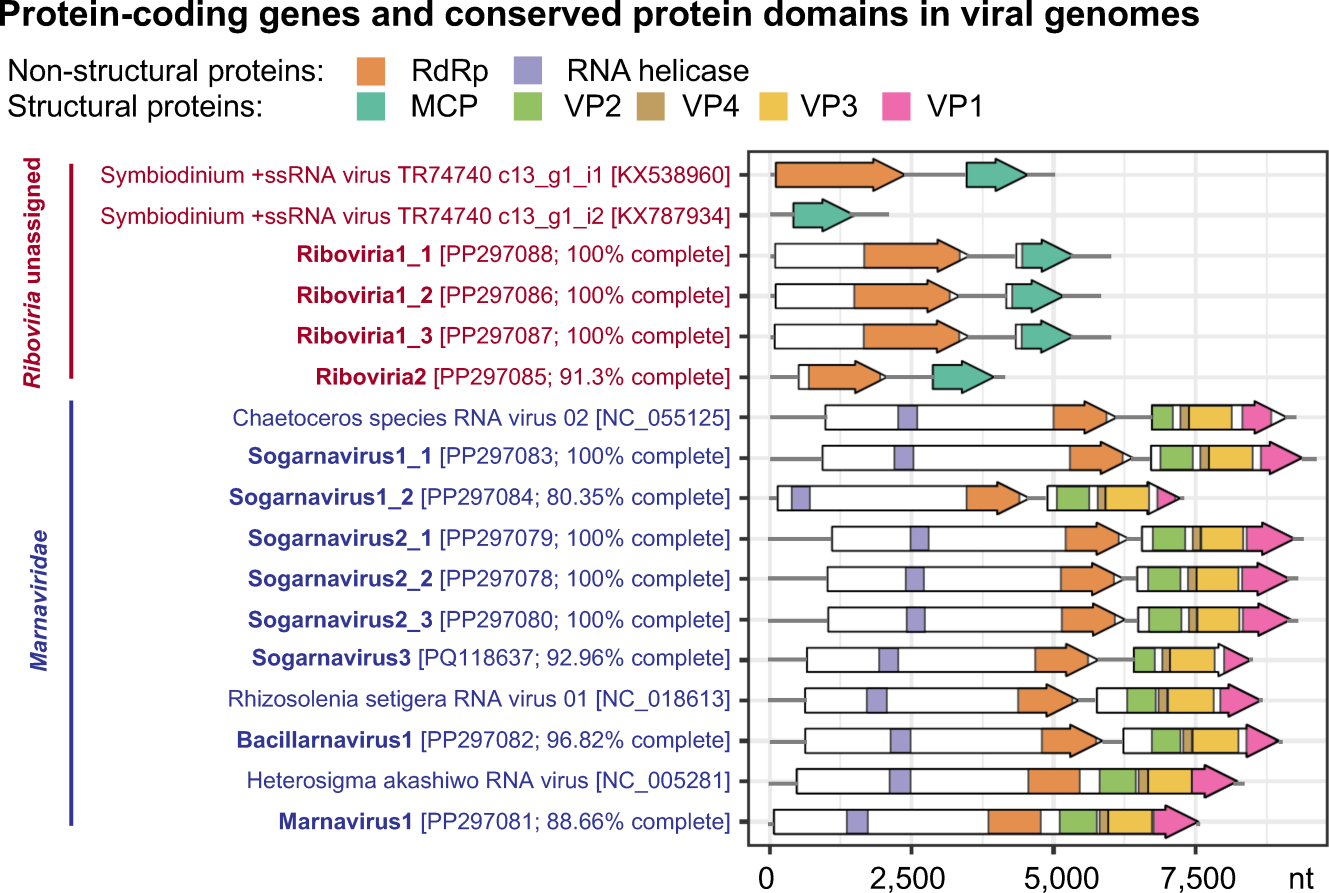
(**A**) Organization of open reading frames (ORFs; arrows) and their protein products (colored arrows) or conserved protein domains (colored subregions within white arrows) in genomes assembled in this study (bold text), in relation to related genomes from the realm unclassified *Riboviria* (red text) and the family *Marnaviridae* (blue text). Square brackets contain NCBI accession numbers for all genomes and predicted % completeness for genomes assembled in this study.

**Fig 3:**
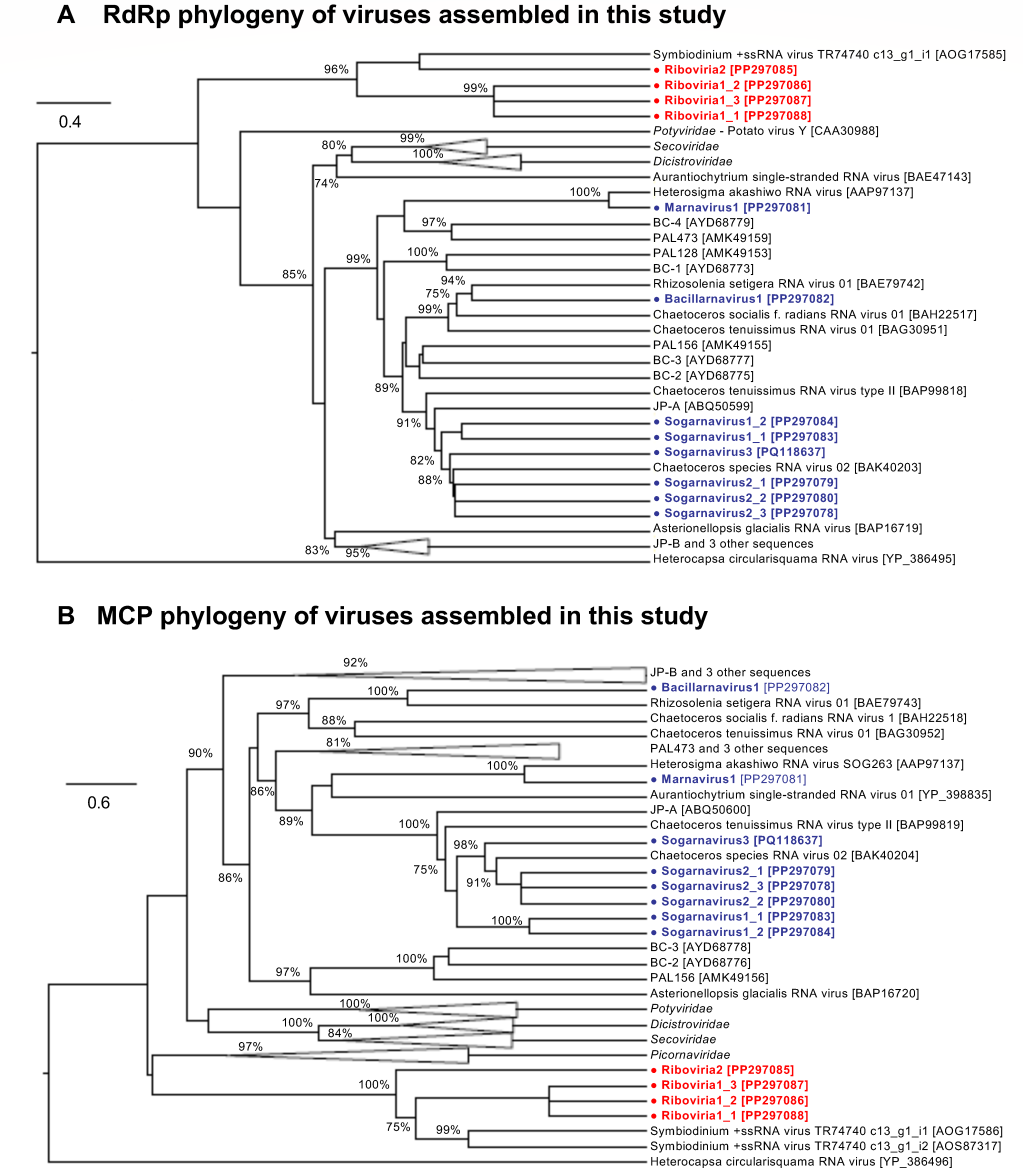
Maximum likelihood tree of (**A**) RdRp and (**B**) MCP amino acid sequences from viral genomes assembled in this study (bold text with round markers), in relation to sequences from related *Picornavirales* and unclassified *Riboviria* genomes. Assembled sequences assigned to unclassified *Riboviria* are highlighted in red, whereas assembled sequences assigned to the order *Picornavirales*, family *Marnaviridae*, and genera *Marnavirus*, *Bacillarnavirus*, and *Sogarnavirus* are highlighted in blue. The RdRp and MCP sequences of Heterocapsa circularisquama RNA virus 01, from the order *Sobelivirales*, were used as the outgroup in panels A and B, respectively. Node labels indicate SH-like branch support values > 70%. The maximum-likelihood scale bar indicates the average number of substitutions per site. NCBI accession numbers for sequences in panels **A** and **B** are indicated in square brackets, where possible, and listed in Data Set S1.

Among the 12 assembled genomes, four genomes were most similar to viruses infecting dinoflagellates that remain unclassified within the *Riboviria*, including Riboviria1_1, Riboviria1_2, Riboviria1_3, and Riboviria2. (Table 1). The four genomes contained two open reading frames (ORFs) each encoding the RNA-dependent RNA polymerase (RdRp) and the major capsid protein (MCP; Fig. 2). Based on CheckV’s (36) estimates, genomes Riboviria1_1, Riboviria1_2, and Riboviria1_3 were complete with an average genome size of 6,012 ± 97 nt, whereas genome Riboviria2 was ~91% complete and 4,158 nt in length (Table 1). Genomes Riboviria1_1, Riboviria1_2, and Riboviria1_3 shared 99% ± 1% pairwise nucleotide identity, 97% ± 2% RdRp sequence identity, and 100% capsid protein sequence identity with each other, but <60% pairwise nucleotide identity, <35% RdRp protein sequence identity, and 77% capsid protein identity to the genome Riboviria2 (Data Set S1). Based on their RdRp and capsid protein phylogeny (Fig. 3), the genomes Riboviria1_1, Riboviria1_2, and Riboviria1_3 were related to Symbiodinium + ssRNA virus TR74740 (NCBI accessions: KX538960 and KX787934) (63), with which they shared 43% ± 6% RdRp sequence identity, but 80% ± 1% capsid protein sequence identity (Data Set S1). The *Riboviria* genomes assembled in this study shared 22% ± 3% RdRp sequence identity and 45% ± 0% capsid protein sequence identity to the genome of Heterocapsa circularisquama RNA virus 01 (HcRNAV; NCBI accession: NC_007518) that infects the HAB-forming dinoflagellate species *Heterocapsa circularisquama* (64).

The remaining eight genomes assembled in this study were most similar to phytoplankton-infecting viruses in the order *Picornavirales* and the family *Marnaviridae* (Table 1). Genera in this family are defined by the International Committee on Taxonomy of Viruses (ICTV) based on phylogenetic relationships of their RdRp protein sequences, whereas species are demarcated by a 90% RdRp sequence identity and 75% capsid protein sequence identity threshold (45). Our phylogenetic analysis assigned six assembled genomes to the genus *Sogarnavirus* and one genome each to the genera *Bacillarnavirus* and *Marnavirus* (Fig. 3).

The sogarnaviruses are represented by the complete 9,790-nt genome, Sogarnavirus1_1, and partial (~80%) 7,225-nt genome, Sogarnavirus1_2. The genomes shared 78% pairwise nucleotide identity, 99% RdRp sequence identity, and 88% capsid sequence identity with each other (Table 1; Data Set S1). Neither genome could be conclusively assigned to a species because they shared 77±5% capsid protein sequence identity, but <90% RdRp sequence identity with the Chaetoceros species RNA virus 02 (CspRNAV02) genome (NCBI accession: NC_055125; unpublished), which is their closest relative (Data Set S1). The remaining sogarnavirus genomes, Sogarnavirus2_1, Sogarnavirus2_2, Sogarnavirus2_3, and Sogarnavirus3, were assigned to the species *Chaetarnavirus 2*, based on their 99±1% RdRp sequence identity and 85% ± 3% capsid protein sequence identity to the CspRNAV02 genome. The complete genomes Sogarnavirus2_1, Sogarnavirus2_2, and Sogarnavirus2_3 had an average size of 9,600 ± 45 nt (Table 1). They shared identical RdRp and capsid protein sequences and 99% ± 0.6% pairwise nucleotide identity with each other, as well as 97% RdRp sequence identity, 79% capsid protein sequence identity, and 79% ± 0.6% pairwise nucleotide identity with sequences in the Sogarnavirus3 genome, which was 8,488 nt long and ~93% complete (Table 1; Data Set S1).

The genome assigned to the *Bacillarnavirus* genus, Bacillarnavirus1, was 8,983 nt long and ~97% complete (Table 1). This genome was assigned to the species Rhizosolenia setigera RNA virus 01, based on its 96% RdRp sequence identity and 83% capsid protein sequence identity to the Rhizosolenia setigera RNA virus 01 (RsRNAV01) reference genome (NCBI accession: NC_018613) (65). All genomes from the genera *Sogarnavirus* and *Bacillarnavirus* have di-cistronic genome organizations (Fig. 2), where the first ORF encodes non-structural proteins with the RNA helicase and RdRp domains, while the second ORF encodes the structural proteins VP2, VP4, VP3 and VP1 conserved in the family *Marnaviridae* (45).

Marnavirus1, the only genome representing the *Marnavirus* genus, was 7,710 nt long and ~89% complete (Table 1). The species of this genome was unassigned because its RdRp and capsid protein domain sequences were 86% and 94% identical, respectively, to the Heterosigma akashiwo RNA virus (HaRNAV) reference genome (NCBI accession: NC_005281) (40). Similar to the HaRNAV genome, genome Marnavirus1 has a mono-cistronic genome organization and encodes a single polyprotein with RNA helicase, RdRp, VP2, VP4, VP3, and VP1 protein sequence signatures (Fig. 2).

### Read coverage and variability

Most viral genomes assembled in this study were only detected by sequencing in library Pool2, which included samples collected from the greater Tampa Bay region during June–July 2021 (Fig. S1). The only exception is the representative genome Sogarnavirus1_1, which was detected in both library Pool1 containing samples from the greater Charlotte Harbor region collected during April 2021, as well as library Pool2 (Fig. S1). Among all individually sequenced libraries from Pool2, the representative genomes Riboviria1_1, Riboviria2, Sogarnavirus1_1, Sogarnavirus3, and Bacillarnavirus1 had the highest coverage in library Pool2_13 (Fig. S1). This library was sequenced from a surface water sample collected at Boca Ciega Bay in June 2021 with ~1.3 million *K*. *brevis* cells/L (Table S1). The highest coverage for the representative genomes Sogarnavirus2_1 and Marnavirus1 was in library Pool2_14 (Table S1; Fig. S1). This library was sequenced from a surface water sample collected from Bayboro Harbor in June 2021 and estimated to contain ~0.4 million *K*. *brevis* cells/L (Table S5).

The mean variability of the representative genomes, measured as the number of single nucleotide variants per kilobase, was generally positively correlated to coverage values. Most representative genomes had higher mean variability values than their coverage values, except for genomes Riboviria1_1, Sogarnavirus 1_1, and Sogarnavirus2_1 (Fig. S1). The representative genomes Riboviria1_1 and Riboviria2 showed higher site entropy in the MCP coding region compared with the RdRp coding region (Fig. S2). In contrast, the other representative genomes classified to the family *Marnaviridae* showed higher site entropy in the RdRp and capsid protein coding regions compared to the RNA helicase protein-coding region (Fig. S2).

### RT-PCR assay design

Specific primer pairs (Table S2) were designed to develop reverse-transcriptase PCR (RT-PCR) assays for each representative viral genome (Table 1). All newly designed primers were initially tested on the 11 samples from Pool1 and Pool2 used for virome sequencing. Four of the seven primer pairs yielded PCR products from at least one Pool1 sample, whereas all seven primer pairs yielded PCR products from at least one Pool2 sample (Table S3). Sequences from the genomes Riboviria2, Bacillarnavirus1, and Marnavirus1 were not amplified in any Pool1 samples (Table S3). Overall, these primers showed higher sensitivity in detecting target sequences compared with virome sequencing. All viral genomes sequenced in the virome libraries (Fig. S1) were also successfully amplified by their genome-specific primers (Table S3). Furthermore, RT-PCR detected viral sequences in Pool1 and Pool2 samples that were not detected previously by virome sequencing (Fig. S1; Table S3).

### Virus detection

**Fig 4:**
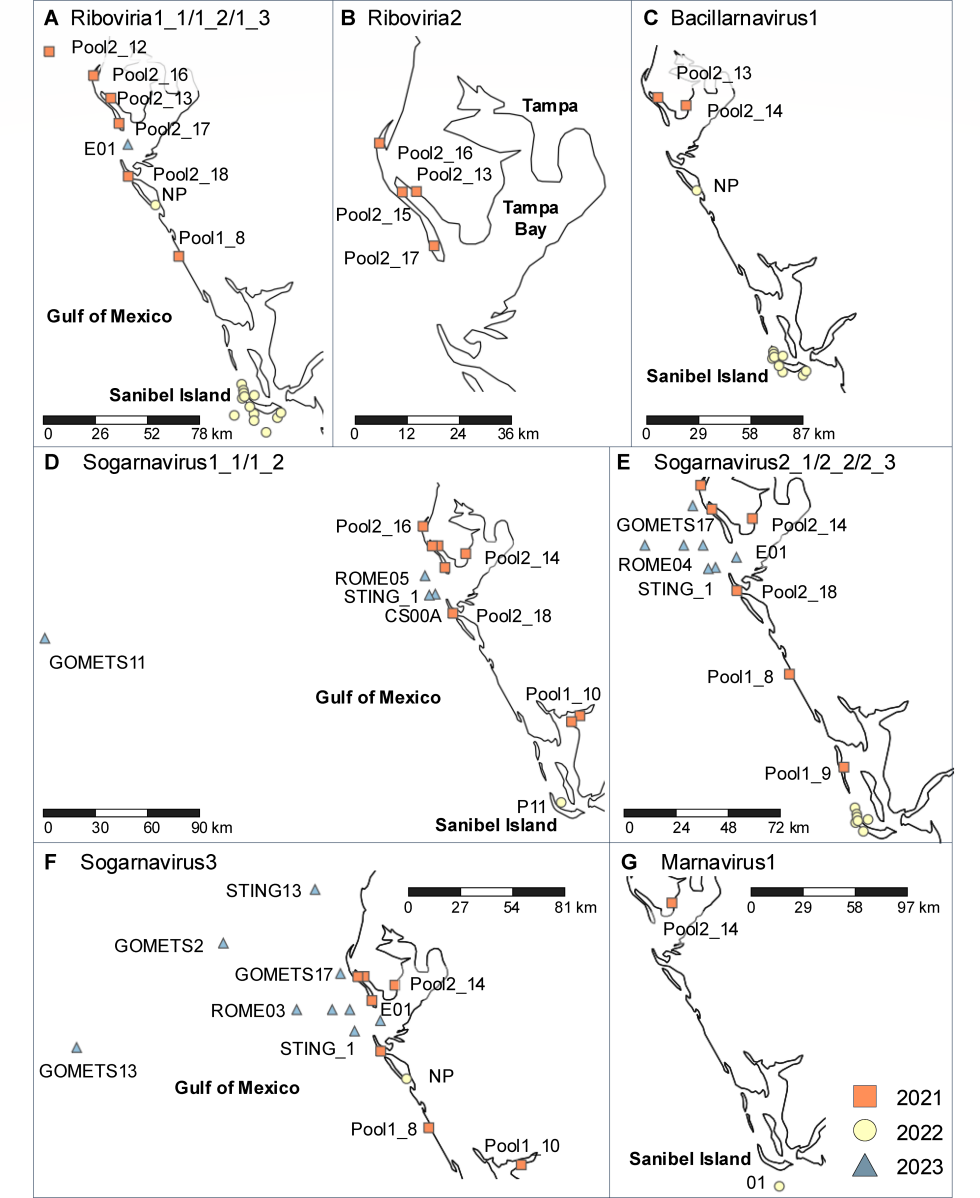
Map of locations where sequence fragments were amplified from the genomes (**A**) Riboviria1_1, Riboviria1_2, and Riboviria1_3; (**B**) Riboviria2; (**C**) Bacillarnavirus1; (**D**) Sogarnavirus1_1 and Sogarnavirus1_2; (**E**) Sogarnavirus2_1, Sogarnavirus2_2, and Sogarnavirus2_3; (**F**) Sogarnavirus3; and (**G**) Marnavirus1. Marker shapes and colors correspond to the year of sample collection. Metadata for collected seawater samples is described in Tables S1 and S6.

Using these primers, RT-PCR assays were performed to screen for the presence of viral genomes in seawater samples collected during a more typical *K. brevis* bloom event documented from October 2022 to May 2023 (62). The screening included 43 additional samples collected at varying depths from 28 locations around southwest Florida between November 2022 and May 2023 (Table S1). For viral diversity analysis, presence/absence data from these 43 samples was combined with data from the 11 samples used for virome sequencing. At least one viral genome was detected in all 11 samples collected in 2021, 15 of 16 samples collected in 2022, and 18 of 27 samples collected in 2023 (Table S6). Viruses were detected mainly in nearshore, surface (<3 m depth) water samples (Fig. 4; Table S6). *Sogarnavirus* sp. and *Chaetarnavirus 2*, both from the genus *Sogarnavirus*, had the broadest distribution compared with other ssRNA viruses analyzed in this study. Sequence fragments from these genomes were amplified in 37 near-shore and open ocean samples collected from depths between 0.1 m and 8.8 m (Fig. 4D through F; Table S6). Among viral genomes assigned to unclassified *Riboviria*, the viral taxon represented by Riboviria1 genomes was more widely distributed compared with the viral taxon represented by genome Riboviria2. Conserved sequence fragments from the Riboviria1 genomes were amplified from 22 samples, spanning the north and south of the southwest Florida region, collected from varying depths (0.5 m–2.7 m) and dates (2021–2023) (Fig. 4A; Table S6). In contrast, sequence fragments from the genome Riboviria2 were amplified from only four samples collected at the surface (0.5 m) depth around St. Petersburg between June and July 2021 (Fig. 4B; Table S6). Sequence fragments from the genome Bacillarnavirus1 were detected in 11 seawater samples, including two surface samples (0.5 m depth) collected from St. Petersburg (War Veteran’s Memorial Park and Bayboro Harbor) in June 2021, two samples collected at 0.5 m and 2.7 m depths from New Pass Dock in November 2022, and seven surface samples (0.5 m depth) collected from Sanibel Island in November 2022 (Fig. 4C; Table S6). *Marnavirus* sp. was the least prevalent in the seawater samples, since sequence fragments from the genome Marnavirus1 were only amplified from two samples, including Pool2_14 from Bayboro Harbor and sample 01 from Sanibel Island (Fig. 4G).

### Viral diversity

**Fig 5:**
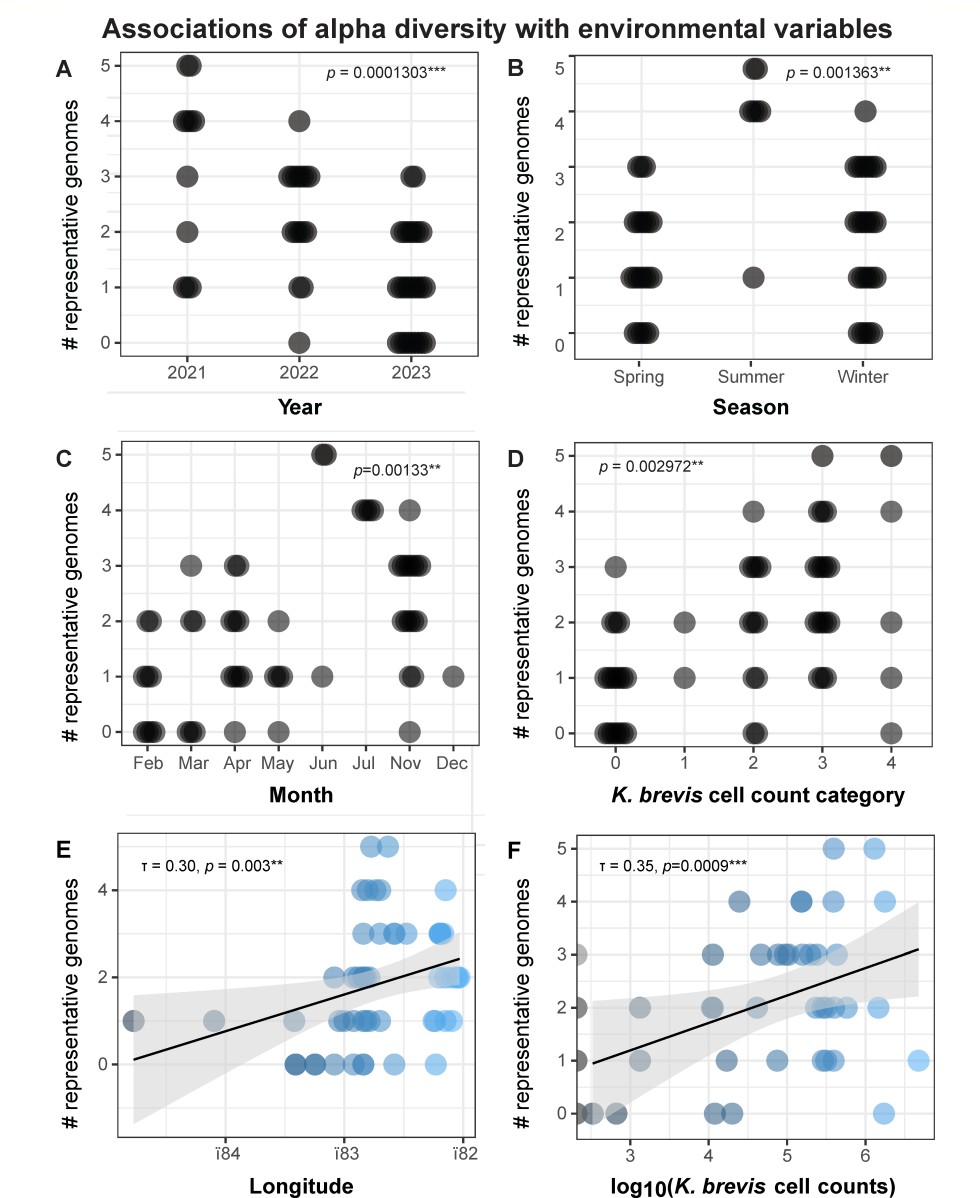
Statistical associations of alpha diversity with environmental variables, including (**A**) year; (**B**) season; (**C**) month; (**D**) *K. brevis* cell count category, (**E**) longitude; and (**F**) *K. brevis* cell counts (cells/L) represented as log_10_(*K. brevis* cell counts). Each marker represents a seawater sample and markers in panels **A–D** are arranged in beeswarm plots to reduce overlap. Marker colors correspond to the categories (**A–D**) or numerical values (**E and F**) of environmental variables. *P*-values in panels **A–D** are determined using Kruskal-Wallis tests, whereas Kendall’s τ correlation coefficient and *P*-values are reported in panels **E and F**. ** denotes a *P*-value of ≤0.01 and *** denotes a *P*-value of ≤0.001.

Alpha diversity, measured as the number of representative genomes from each taxon detected in each sample using RT-PCR, was significantly different between years, seasons, and months. Samples collected in 2021 during the prolonged *K. brevis* bloom event, and during the time of year when most blooms have subsided, had the highest alpha diversity, followed by samples collected in 2022 and then 2023, which represented the earlier and later parts of the 2022–2023 bloom event, respectively (Fig. 5A). Alpha diversity was the highest in samples collected during the summer months, compared with samples collected during the winter and spring months (Fig. 5B and C). Alpha diversity was also positively correlated with longitude (Fig. 5E), but not with latitude and depth. Additionally, alpha diversity was significantly different across *K. brevis* cell count categories (Fig. 5D), reflecting a positive correlation with *K. brevis* cell counts (Fig. 5F). To further investigate relationships between these predictor variables, we performed multiple regression using alpha diversity as the response variable. The optimum model (adjusted *R*^2^ = 0.4945, *P* = 0.00001538) contained two predictor variables, including month (*P* = 0.002) and *K. brevis* cell count category (*P* = 0.0348).

**Fig 6:**
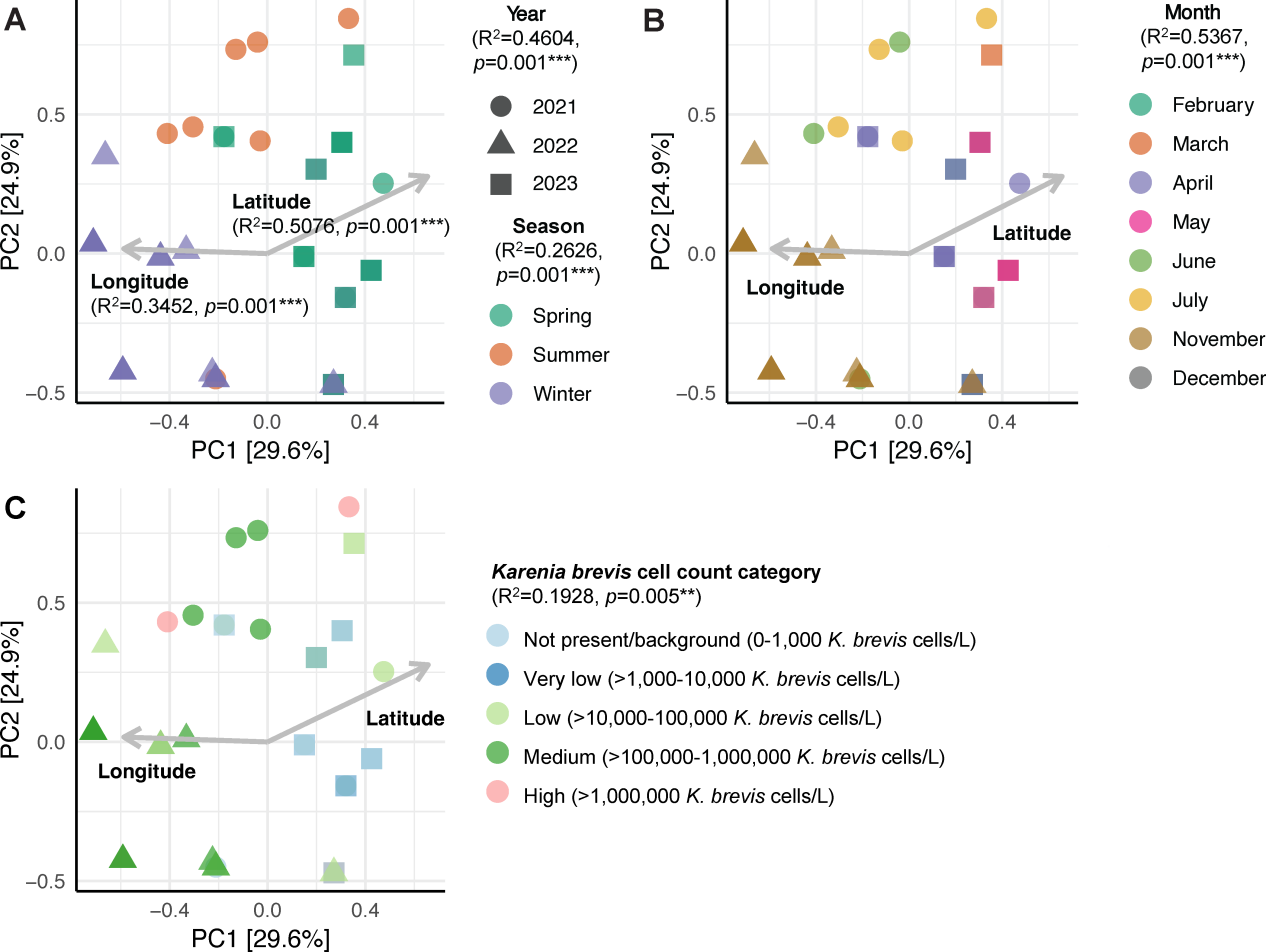
Principal components analysis (PCA) biplot showing the significant effects of longitude; latitude; (**A**) year and season; (**B**) month; and (**C**) *K. brevis* cell count category on the clustering of all field-collected samples. Each point in the PCA biplot represents a seawater sample and marker shapes correspond to the year of sample collection. Percentage values in square brackets on each axis represent the relative contribution (eigenvalue) of each axis to the total inertia (variation) in the data set. The goodness-of-fit *R*^2^ value and *P*-value for each variable are shown on the biplots. ** denotes a *P*-value of ≤0.01 and *** denotes a *P*-value of ≤0.001.

Beta diversity was significantly correlated with latitude, longitude, year, season, month, and *K. brevis* cell count category (Fig. 6). Multiple regression using *K. brevis* cell counts as the response variable generated an optimum model (adjusted *R*^2^ = 0.7887, *P* = 8.035 × 10^−13^) with two predictor variables, including month (*P* = 0.026) and the presence of sequences amplified from the genome Riboviria2 (*P* = 0.039).

To explore whether ssRNA viruses identified from field samples in this study could also be associated with cultivated *Karenia* species, we performed RT-PCR assays on *Karenia* spp. cell cultures routinely maintained in the laboratory at FWRI, including 17 *K*. *brevis* strains (CCFWC 242, 254, 257, 258, 261, 267, 121, 123, 124, 125, 126, 1010, 1012, 1013, 1014, 1016, and 1021), *K. mikimotoi* strain CCFWC 67, *K. papilionacea* strain CCFWC 1020, and *K. umbella* strain 1019 B2 (Table S4). These samples did not yield visible PCR products with any primer pair.

## DISCUSSION

In this study, we recovered complete or near-complete genomes of ssRNA viruses from seawater collected around southwest Florida during a long *K. brevis* bloom event that began in 2020 and lasted throughout most of 2021 (62). We developed targeted RT-PCR assays for these genomes to examine their occurrence and ecology in additional seawater samples collected in the region during a subsequent and shorter *K. brevis* bloom event between October 2022 and May 2023 (62).

Most of the assembled viral genomes belonged to the order *Picornavirales* and family *Marnaviridae* (45). Genomes assigned to the genus *Sogarnavirus* and *Bacillarnavirus* exhibit synteny and significant RdRp and capsid sequence similarity to genomes of viral species known to infect bloom-forming diatoms. These include Chaetarnavirus 2 (CspRNAV02), which infects *Chaetoceros* sp., and Rhizosolenia setigera RNA virus 01 (RsRNAV01), which infects *R. setigera* (65). On the other hand, the genome assigned to the genus *Marnavirus* was related to Heterosigma akashiwo RNA virus (HaRNAV), which infects the HAB-forming raphidophyte species *Heterosigma akashiwo* (40). The prevalence of these ssRNA viruses, particularly *Sogarnavirus* sp., in our field-collected samples, is not surprising, given that these phytoplankton genera are commonly observed in southwest Florida (FWC HAB Monitoring Database). *Chaetoceros* sp., *R. setigera*, and *H. akashiwo* are globally distributed (66–68) and were previously identified in a 16S rRNA gene-based metabarcoding survey of seawater samples collected in Tampa Bay during the June 2018 *K. brevis* bloom (69). *H. akashiwo* has also been isolated from Tampa Bay (70, 71). *Chaetoceros* sp. and *H. akashiwo* are known HAB species that are associated with fish kills, especially at salmonid aquaculture net pens in Washington State (72, 73). Diatom species from the *Chaetoceros* and *Rhizosolenia* genera (including *R. setigera*) contain spines that could physically damage fish gills through clogging, mechanical irritation, puncturing, and/or secondary bacterial infections (68, 73). Although *R. setigera* is generally not considered a HAB species, there have been reports of fish mortalities caused by *R. setigera* blooms in Canada (74) and China (75). Algal viruses typically exhibit intraspecies host specificity with variations in strain specificity (76, 77). In contrast, an algal species can be infected by multiple strains of a virus, and strains of an algal species can differ in virus susceptibility (76, 77). Currently, the nature and impacts of phytoplankton-phytoplankton and phytoplankton-microbial interactions on *K. brevis* blooms in Florida are poorly understood. Although phytoplankton, bacterial, and archaeal community composition was investigated during the June 2018 bloom (69), our study provides the first analysis of the diversity of RNA viruses associated with *K. brevis* blooms and suggests that like blooms of *K. brevis*, associated viral assemblages are complex and dynamic.

In addition to genomes belonging to the *Marnaviridae* family, we also recovered genomes assigned to unclassified *Riboviria*. These genomes likely represent new viral taxa that are related to non-retroviral dinoflagellate-infecting +ssRNA virus (dinoRNAV) prevalent in coral holobionts (63, 78, 79) and distantly related to HcRNAV infecting the HAB-forming dinoflagellate species *Heterocapsa circularisquama* (64). Similar to dinoRNAV (63, 79) and HcRNAV (64), the unclassified *Riboviria* genomes contain two ORFs encoding RdRp and MCP. DinoRNAV has not been isolated in culture, but meta’omics analysis suggested that these viruses are endogenized within the genomes of coral dinoflagellate symbionts belonging to the family Symbiodiniaceae, particularly the genus *Symbiodinium* (64). The unclassified *Riboviria* genomes assembled in this study were most closely related to a dinoRNAV genome sequenced from a thermosensitive type C1 *Symbiodinium* culture (63). Furthermore, transcript expression of this dinoRNAV was downregulated under heat stress (63). In contrast, other dinoRNAV taxa were associated with heat-stressed coral samples (78) or showed higher MCP sequence diversity under heat stress (80). Because of their differential expression and diversity under thermal stress conditions, dinoRNAV are hypothesized to contribute to symbiont colonization and coral bleaching, which is partly driven by climate change (80). Although the presence of the genome Riboviria2 was a significant predictor of *K. brevis* cell counts, the small and imbalanced sample size in this study precluded statistical analysis of whether *K. brevis* cell counts could predict the presence (*n* = 4) or absence (*n* = 50) of sequences from the genome Riboviria2. Furthermore, the observation of Riboviria2 only during the summer months of 2021, outside the typical bloom season, is intriguing; further sampling can help evaluate whether Riboviria2 occurs seasonally even if additional blooms do not persist through summer. We were also unable to determine the host of these unclassified *Riboviria* species. RT-PCR screening on *Karenia* spp. (*K. brevis*, *K. mikimotoi*, *K. papilionacea*, and *K. umbella*) cultures maintained in the laboratory over extended time periods did not amplify sequence fragments from these unclassified *Riboviria* genomes. Future culturing, imaging, and single-cell RNA sequencing experiments on fresh seawater samples containing *K. brevis* blooms are critical to isolate, characterize, and identify the host species of the unclassified *Riboviria* and other ssRNA viruses identified in this study. Since temperature data were not recorded for all our seawater samples, we were also unable to explore the effects of temperature on the prevalence or abundance of these unclassified *Riboviria* taxa and other viral taxa identified in this study beyond seasonal climatology (81). Notably, month was a significant predictor of both RNA viral diversity and *K. brevis* cell counts, and higher viral diversity was observed during the bloom event that persisted into the summer. Temporal effects could correspond to environmental parameters, including temperature, salinity, light availability, and nutrient concentrations, which have been known to influence the growth rate and physiology of *K. brevis* (82) and the seasonality of *K. brevis* blooms (83).

Overall, our study provided valuable information on the diversity of ssRNA viruses associated with *K. brevis* blooms and unveiled new *Riboviria*-dinoflagellate associations in the waters of southwest Florida. Viruses play important roles in phytoplankton dynamics and cell death that can influence bloom dynamics (24). Although viral interactions with other phytoplankton species are relatively well-studied (69, 84–87), viruses infecting *K. brevis* and co-occurring phytoplankton species during bloom events have been poorly investigated. Long-term surveillance of *K. brevis* blooms and their microbial and viral community composition, coupled with mechanistic studies, will help elucidate the roles of viruses in bloom ecology and their responses to environmental factors, including climate change, in this region.

## Data Availability

Sequenced reads and viral genomes were deposited in NCBI under the BioProject accession number PRJNA1149755. Viral genomes were assigned the GenBank accession numbers PP297078-PP297088 and PQ118637.

## SUPPLEMENTAL MATERIAL

Data Set S1 msphere.01090-24-s0001.xlsx ANI and AAI.

Figure S1 msphere.01090-24-s0002.tiff Mean coverage.

Figure S2 msphere.01090-24-s0003.tiff Site entropy.

Legends msphere.01090-24-s0004.docx Legends for Figures S1 and S2.

Supplemental Tables msphere.01090-24-s0005.docx Tables S1 to S6.

ASM does not own the copyrights to Supplemental Material that may be linked to, or accessed through, an article. The authors have granted ASM a non-exclusive, world-wide license to publish the Supplemental Material files. Please contact the corresponding author directly for reuse.
