# Supplementary figures and images for: Diverse ssRNA viruses associated with Karenia brevis harmful algal blooms in southwest Florida
Source: mSphere. 2025 Mar 20;10(4):e01090-24. doi: 10.1128/msphere.01090-24 (PMC12039238; doi:10.1128/msphere.01090-24)

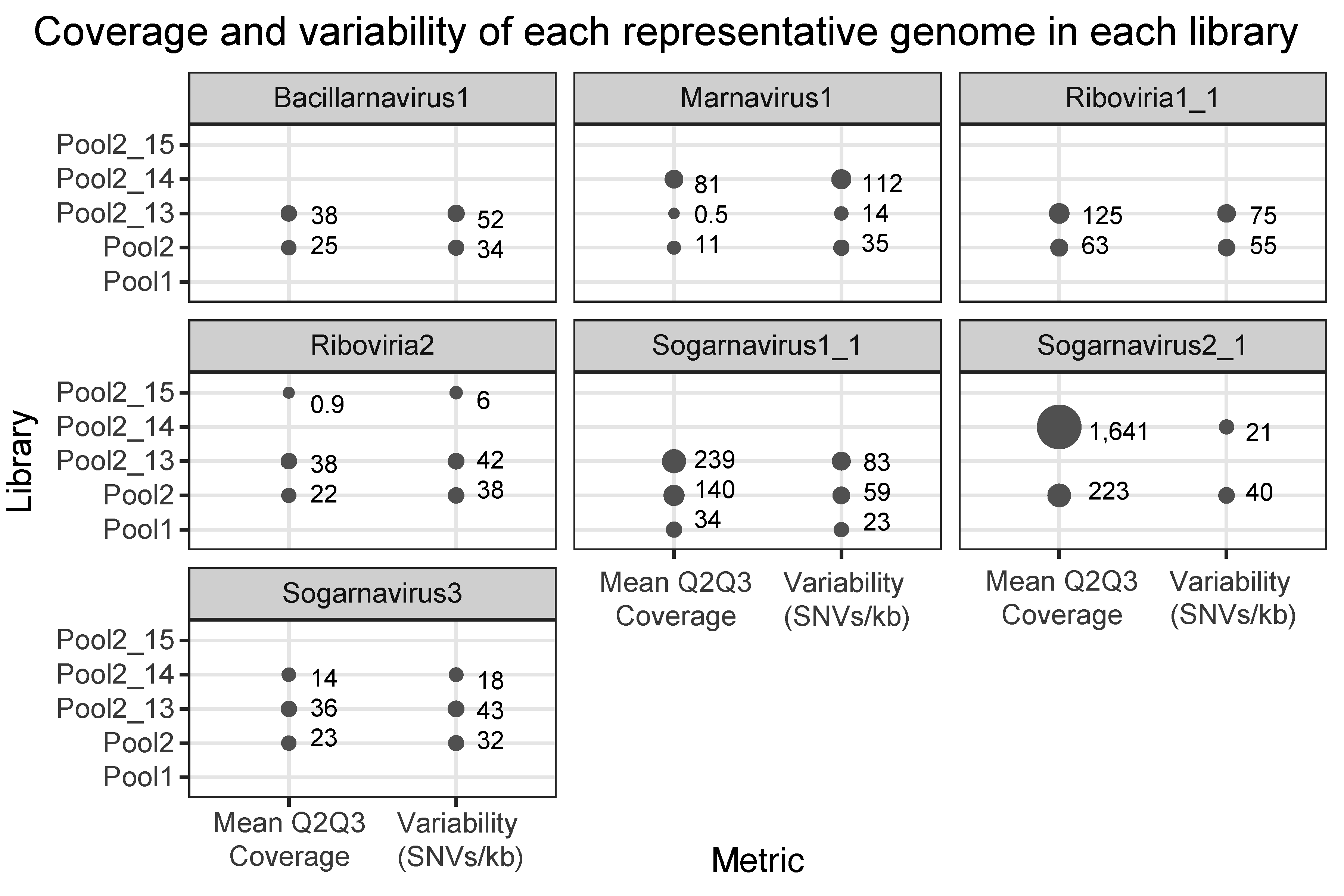

Supplement: Figure S1 — Mean coverage. [file msphere.01090-24-s0002.tiff]

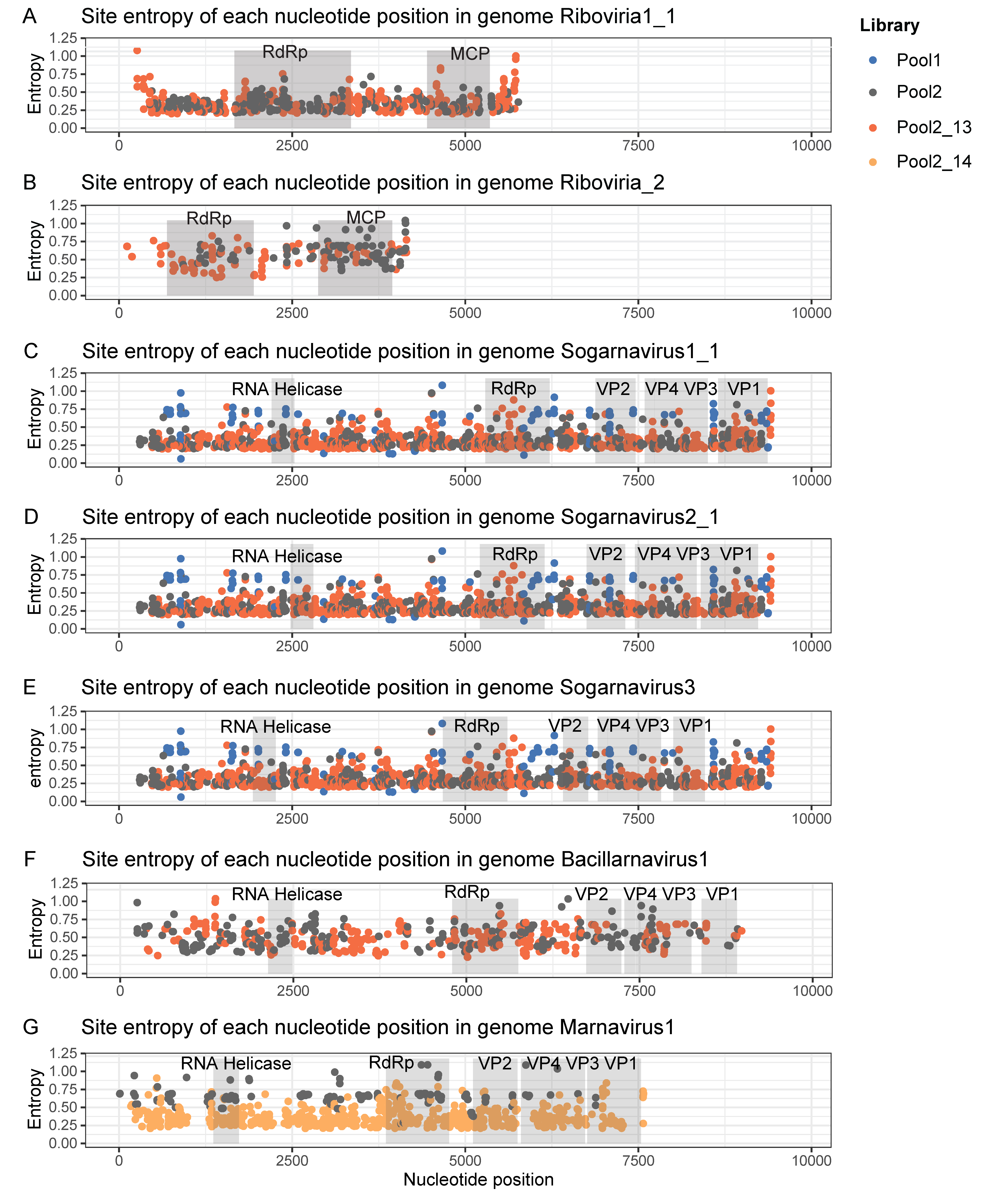

Supplement: Figure S2 — Site entropy. [file msphere.01090-24-s0003.tiff]
