# Supplementary material for: Diverse ssRNA viruses associated with Karenia brevis harmful algal blooms in southwest Florida
Source: mSphere. 2025 Mar 20;10(4):e01090-24. doi: 10.1128/msphere.01090-24 (PMC12039238; doi:10.1128/msphere.01090-24)
Supplement: Legends — for Figures S1 and S2. [file msphere.01090-24-s0004.docx]

**Figure S1.** Mean Q2Q3 coverage and variability, measured as the number of reported single nucleotide variants per kilo base pair (SNVs/kb), of representative genomes from each viral taxon in each sequenced library. Only positions with ≥10 coverage values were included in the variability calculations.

**Figure S2.** Site entropy of each nucleotide position in the representative genomes (a) Riboviria1_1; (b) Riboviria2; (c) Sogarnavirus1_1; (d) Sogarnavirus2_1; (e) Sogarnavirus3; (f) Bacillarnavirus1; and (g) Marnavirus1. Only positions with >0 entropy and non-outlier coverage values compared to all other positions in each genome were shown. Annotated protein-coding regions in each genome are shaded in grey.
