# Supplementary material for: Diverse ssRNA viruses associated with Karenia brevis harmful algal blooms in southwest Florida
Source: mSphere. 2025 Mar 20;10(4):e01090-24. doi: 10.1128/msphere.01090-24 (PMC12039238; doi:10.1128/msphere.01090-24)
Supplement: Supplemental Tables — Tables S1 to S6. [file msphere.01090-24-s0005.docx]

**Table S1.** Metadata of samples analyzed in this study. Samples used for virome sequencing are highlighted in bold. Samples used for pooled libraries are indicated with the prefixes Pool1 and Pool2. Samples from Pool2 that were subsequently sequenced individually are indicated with *. *Karenia brevis* cell counts, available from the FWC HAB Monitoring Database (<https://geodata.myfwc.com/datasets/myfwc::recent-harmful-algal-bloom-hab-events>), were matched to the exact or closest (indicated with ^ in the last column) sample collection date, depth, latitude, and longitude. Metadata of samples used for *K. brevis* counts are provided in **Table S5**.

| Sample ID | Collection Date | Location | Latitude | Longitude | Depth (m) | *K. brevis* (cells/L) |
| --- | --- | --- | --- | --- | --- | --- |
| Pool1_8 | 4/6/2021 | Venice | 27.1091 | -82.4773 | 0.5 | 46,500^ |
| Pool1_9 | 4/6/2021 | Boca Grande | 26.7188 | -82.2488 | 0.5 | 17,000^ |
| Pool1_10 | 4/29/2021 | Laishley Park – boat ramp | 26.93939 | -82.052278 | 0.5 | 11,000 |
| Pool1_11 | 4/29/2021 | Ponce de Leon Park – boat ramp | 26.90927 | -82.09481 | 0.1 | 285,311 |
| Pool2_12* | 6/13/2021 | Gulf of Mexico – edge of offshore high Chl-a patch | 28.00568 | -83.04386 | 1 | 0^ |
| Pool2_13* | 6/17/2021 | War Veteran's Memorial Park | 27.80192 | -82.7746 | 0.5 | 1,298,692 |
| Pool2_14* | 6/30/2021 | Bayboro Harbor | 27.761897 | -82.6329 | 0.5 | 393,333^ |
| Pool2_15* | 7/25/2021 | Archibald Beach Park | 27.8009 | -82.8034 | 0.5 | 1,769,218 |
| Pool2_16* | 7/25/2021 | Indian Rocks Beach | 27.9006 | -82.8499 | 0.5 | 152,667 |
| Pool2_17* | 7/25/2021 | Pass-A-Grille Beach | 27.69141 | -82.7386 | 0.5 | 390,333 |
| Pool2_18* | 7/26/2021 | W of Cortez Beach (10th Street Pier) | 27.4595 | -82.6982 | 0.5 | 152,667 |
| NP1 | 11/7/2022 | New Pass Dock (Sarasota Bay) | 27.33375 | -82.57938 | 0.5 | 431,030 |
| NP2 | 11/14/2022 | New Pass Dock (Sarasota Bay) | 27.33375 | -82.57938 | 2.7 | 76,667 |
| NP3 | 5/8/2023 | New Pass Dock (Sarasota Bay) | 27.33375 | -82.57938 | 2 | 667 |
| ROME01_022423_surface | 2/24/2023 | 41 mi W of Bunces Pass | 27.64966 | -83.410366 | 0.5 | 0 |
| ROME01_032723_surface | 3/27/2023 | 41 mi W of Bunces Pass | 27.64966 | -83.410366 | 0.5 | 0 |
| ROME02_022423_surface | 2/24/2023 | 31 mi W of Bunces Pass | 27.64966 | -83.2474 | 0.5 | 0 |
| ROME02_032723_surface | 3/27/2023 | 31 mi W of Bunces Pass | 27.64966 | -83.2474 | 0.5 | 0 |
| ROME03_022423_surface | 2/24/2023 | 21 mi W of Bunces Pass | 27.64966 | -83.084434 | 0.5 | 1,333 |
| ROME03_032723_surface | 3/27/2023 | 21 mi W of Bunces Pass | 27.64966 | -83.084434 | 0.5 | 333 |
| ROME04_120522_surface | 12/5/2022 | 11 mi W of Bunces Pass | 27.64966 | -82.921468 | 0.5 | 4,752,180 |
| ROME04_022423_surface | 2/24/2023 | 11 mi W of Bunces Pass | 27.64966 | -82.921468 | 0.5 | 12,000 |
| ROME04_032723_surface | 3/27/2023 | 11 mi W of Bunces Pass | 27.64966 | -82.921468 | 0.5 | 41,333 |
| ROME05_022423_surface | 2/24/2023 | 5.8 mi W of Bunces Pass | 27.64966 | -82.84019 | 0.5 | 1,719,010 |
| ROME05_022423_bottom | 2/24/2023 | 5.8 mi W of Bunces Pass | 27.64966 | -82.84019 | 8.8 | 1,333 |
| ROME05_032723_surface | 3/27/2023 | 5.8 mi W of Bunces Pass | 27.64966 | -82.84019 | 0.5 | 94,000 |
| ROME05_032723_bottom | 3/27/2023 | 5.8 mi W of Bunces Pass | 27.64966 | -82.84019 | 8.8 | 11,333 |
| ROME05_042523_surface | 4/25/2023 | 5.8 mi W of Bunces Pass | 27.64966 | -82.84019 | 0.5 | 0 |
| ROME05_042523_bottom | 4/25/2023 | 5.8 mi W of Bunces Pass | 27.64966 | -82.84019 | 8.7 | 0 |
| Sanibel_01 | 11/17/2022 | 5.8 mi S of Tarpon Bay Road Beach | 26.33856 | -82.09553 | 0.5 | 576,810 |
| Sanibel_04 | 11/17/2022 | 2.4 mi SE of Tarpon Bay Road Beach | 26.40238 | -82.04633 | 0.5 | 1,462,680 |
| Sanibel_B14 | 11/17/2022 | 2.1 mi SW of Lighthouse Beach Park | 26.42421 | -82.03075 | 0.5 | 314,167 |
| Sanibel_D2 | 11/17/2022 | 4.5 mi S of Bowmans Beach | 26.3897 | -82.14855 | 0.5 | 75,000 |
| Sanibel_P11 | 11/17/2022 | 1.2 mi E of Chino Island (Pine Island Sound) | 26.500267 | -82.1473 | 0.5 | 24,667 |
| Sanibel_D14 | 11/17/2022 | 2.5 mi S of Bowmans Beach | 26.41931 | -82.14855 | 0.5 | 234,125 |
| Sanibel_E1 | 11/17/2022 | SW of Bowmans Beach | 26.45062 | -82.16691 | 0.5 | 11,333 |
| Sanibel_E2A | 11/17/2022 | 5.8 mi SW of Bowmans Beach | 26.41249 | -82.23468 | 0.5 | 392,551 |
| Sanibel_F1 | 11/17/2022 | 1 mi NW of Blind Pass | 26.48713 | -82.19962 | 0.5 | 104,055 |
| Sanibel_F2 | 11/17/2022 | 3.1 mi W of Blind Pass | 26.46784 | -82.23019 | 0.5 | 20,000 |
| Sanibel_G503 | 11/17/2022 | SW of Redfish Pass | 26.54911 | -82.20238 | 0.5 | 283,667 |
| Sanibel_G506 | 11/17/2022 | SW of Alison Hagerup Beach | 26.52273 | -82.19497 | 0.5 | 157,284 |
| Sanibel_G507 | 11/17/2022 | W of Captiva Island Yacht Club | 26.50999 | -82.19332 | 0.5 | 239,667 |
| Sanibel_G508 | 11/17/2022 | W of Osprey Way Drive | 26.49495 | -82.19012 | 0.5 | 196,905 |
| STING_1_1m | 2/20/2023 | STING Station 1 - CTD | 27.5515 | -82.8184 | 1 | 398,000^ |
| STING_1_8m | 2/20/2023 | STING Station 1 - Pump | 27.5515 | -82.8184 | 8 | 315,000^ |
| STING_13_2m | 2/26/2023 | STING Station 13 | 28.2006 | -83.0001 | 2 | 0^ |
| E01 Surface | 4/25/2023 | 1.8 mi S of Mullet Key (Lower Tampa Bay) | 27.5991 | -82.7 | 0.5 | 0 |
| E01 Bottom | 4/25/2023 | 1.8 mi S of Mullet Key (Lower Tampa Bay) | 27.5991 | -82.7 | 5.9 | 0 |
| CS00A Surface | 4/25/2023 | 2.3 mi SW of Egmont Key | 27.55661 | -82.78781 | 0.5 | 0 |
| CS00A Bottom | 4/25/2023 | 2.3 mi SW of Egmont Key | 27.55661 | -82.78781 | 7.2 | 0 |
| GOMETS2 | 5/8/2023 | GOMETS Station 2 | 27.95464 | -83.42188 | 1 | 0^ |
| GOMETS11 | 5/12/2023 | GOMETS Station 11 | 27.3325 | -84.7740 | 1 | 0^ |
| GOMETS13 | 5/12/2023 | GOMETS Station 13 | 27.4762 | -84.0963 | 1 | 0^ |
| GOMETS17 | 5/13/2023 | GOMETS Station 17 | 27.8153 | -82.8835 | 1 | 0^ |

**Table S2.** List of PCR primers used in this study.

| **Primer Name** | **Genome target** | **Gene Product** | **Sequence (5'->3')** | **Amplicon Size** | **Annealing Temperature** |
| --- | --- | --- | --- | --- | --- |
| Riboviria1_RdRp_F | Riboviria1_1, Riboviria1_2, Riboviria1_3 | RNA-dependent RNA polymerase | CAGATCCACGCCTCATATTC | 571 bp | 54.8℃ |
| Riboviria1_RdRp_R |  |  | GTGAAAGACCAGTGTCGATAG |  |  |
| Riboviria2_Capsid_F | Riboviria2 | Capsid protein | CACCTCATCGACGTTTACAG | 278 bp | 55.4℃ |
| Riboviria2_Capsid_R |  |  | CTGCACATCCCTCGAAATTA |  |  |
|  |  |  |  |  |  |
| Sogarnavirus1_RdRp_F | Sogarnavirus1_1, Sogarnavirus1_2 | RNA-dependent RNA polymerase | CGGAAACCACGCTTATCT | 116 bp | 53.8℃ |
| Sogarnavirus1_RdRp_R |  |  | AGATTCAGAGCAACAGGC |  |  |
| Sogarnavirus2_RdRp_F | Sogarnavirus2_1, Sogarnavirus2_2, Sogaranavirus2_3 |  | AATCGCGCTGCTTACC | 91 bp | 54.3℃ |
| Sogarnavirus2_RdRp_R |  |  | TACCACGACCGGAATCT |  |  |
| Sogarnavirus3_RdRp_F | Sogarnavirus3 |  | GCTCTAGCCGTTACTTTACC | 97 bp | 54.4℃ |
| Sogarnavirus3_RdRp_R |  |  | CGCAAAGGCTACCAGTT |  |  |
|  |  |  |  |  |  |
| Bacillarnavirus1 _RdRp_F | Bacillarnavirus1 |  | CTTTATGATGGTCCCGGTTCTT | 452 bp | 55.1℃ |
| Bacillarnavirus1 _RdRp_R |  |  | CCTGCGCCAGTAAGGTATTT |  |  |
|  |  |  |  |  |  |
| Marnavirus1_VP3_F | Marnavirus1 | Capsid protein (VP3) | CACTTGGTCTCCGTCAAA | 98 bp | 54.4℃ |
| Marnavirus1_VP3_R |  |  | CATACGCATCCGGACTTT |  |  |

**Table S3.** Presence (+) or absence (-) of sequence fragments from each genome in Pool1 and Pool2 samples used for virome sequencing, as determined by RT-PCR. For Pool2 samples, ‘++’ denotes the presence of a RT-PCR product and its corresponding sequence (with >0 mean Q2Q3 coverage) in the virome library (**Table S1**). Pool1 samples were not individually sequenced using viromics and had no sample-specific coverage information.

| **Genome** | Pool1_8 | Pool1_9 | Pool1_10 | Pool1_11 | Pool2_12 | Pool2_13 | Pool2_14 | Pool2_15 | Pool2_16 | Pool2_17 | Pool2_18 |
| --- | --- | --- | --- | --- | --- | --- | --- | --- | --- | --- | --- |
| Riboviria1_1/1_2/1_3 | + | - | - | - | + | ++ | - | - | + | + | + |
| Riboviria2 | - | - | - | - | - | ++ | - | ++ | + | + | - |
| Sogarnavirus1_1/1_2 | - | - | + | + | - | ++ | + | + | + | + | + |
| Sogarnavirus2_1/2_2/2_3 | + | + | - | - | - | - | ++ | + | + | - | + |
| Sogarnavirus3 | + | - | + | - | - | ++ | ++ | + | - | + | + |
| Bacillarnavirus1 | - | - | - | - | - | ++ | + | - | - | - | - |
| Marnavirus1 | - | - | - | - | - | - | ++ | - | - | - | - |

**Table S4.** Metadata of *Karenia* spp. cell culture samples used in the RT-PCR assay.

| **Species** | **Strain ID** | **Replicate** | **Date filtered** | **Volume filtered (mL)** |
| --- | --- | --- | --- | --- |
| Media only | N/A | A | 6/5/2024 | 35 |
| Media only | N/A | B | 6/5/2024 | 35 |
| Media only | N/A | C | 6/5/2024 | 35 |
| *Karenia brevis* | 252 | A | 5/29/2024 | 26 |
| *Karenia brevis* | 252 | B | 5/29/2024 | 20 |
| *Karenia brevis* | 252 | C | 6/5/2024 | 15 |
| *Karenia brevis* | 254 | A | 5/29/2024 | 14 |
| *Karenia brevis* | 254 | B | 5/29/2024 | 14 |
| *Karenia brevis* | 254 | C | 6/5/2024 | 15 |
| *Karenia brevis* | 257 | A | 5/29/2024 | 15 |
| *Karenia brevis* | 257 | B | 6/5/2024 | 15 |
| *Karenia brevis* | 257 | C | 6/5/2024 | 15 |
| *Karenia brevis* | 258 | A | 5/29/2024 | 15 |
| *Karenia brevis* | 258 | B | 5/29/2024 | 14 |
| *Karenia brevis* | 261 | A | 5/29/2024 | 15 |
| *Karenia brevis* | 261 | B | 5/29/2024 | 15 |
| *Karenia brevis* | 261 | C | 6/5/2024 | 15 |
| *Karenia brevis* | 267 | A | 5/29/2024 | 15 |
| *Karenia brevis* | 267 | B | 5/29/2024 | 15 |
| *Karenia brevis* | 267 | C | 6/5/2024 | 15 |
| *Karenia brevis* | 121 | A | 5/29/2024 | 15 |
| *Karenia brevis* | 121 | B | 5/29/2024 | 15 |
| *Karenia brevis* | 121 | C | 6/5/2024 | 15 |
| *Karenia brevis* | 123 | A | 5/29/2024 | 25 |
| *Karenia brevis* | 123 | B | 5/29/2024 | 25 |
| *Karenia brevis* | 123 | C | 6/5/2024 | 15 |
| *Karenia brevis* | 124 | A | 5/29/2024 | 25 |
| *Karenia brevis* | 124 | B | 5/29/2024 | 25 |
| *Karenia brevis* | 124 | C | 6/5/2024 | 15 |
| *Karenia brevis* | 125 | A | 6/5/2024 | 32.5 |
| *Karenia brevis* | 125 | B | 6/5/2024 | 32.5 |
| *Karenia brevis* | 125 | C | 6/12/2024 | 30 |
| *Karenia brevis* | 126 | A | 5/29/2024 | 15 |
| *Karenia brevis* | 126 | B | 5/29/2024 | 15 |
| *Karenia brevis* | 126 | C | 6/5/2024 | 15 |
| *Karenia brevis* | 1010 | A | 5/29/2024 | 15.5 |
| *Karenia brevis* | 1010 | B | 5/29/2024 | 15 |
| *Karenia brevis* | 1010 | C | 6/5/2024 | 15 |
| *Karenia brevis* | 1012 | A | 5/29/2024 | 15 |
| *Karenia brevis* | 1012 | B | 5/29/2024 | 15 |
| *Karenia brevis* | 1012 | C | 6/5/2024 | 15 |
| *Karenia brevis* | 1013 | A | 6/5/2024 | 25 |
| *Karenia brevis* | 1013 | B | 6/5/2024 | 25 |
| *Karenia brevis* | 1013 | C | 6/12/2024 | 25 |
| *Karenia brevis* | 1014 | A | 5/29/2024 | 12 |
| *Karenia brevis* | 1014 | B | 5/29/2024 | 27 |
| *Karenia brevis* | 1014 | C | 6/5/2024 | 25 |
| *Karenia brevis* | 1016 | A | 5/29/2024 | 33 |
| *Karenia brevis* | 1016 | B | 5/29/2024 | 31 |
| *Karenia brevis* | 1016 | C | 6/5/2024 | 25 |
| *Karenia brevis* | 1021 | A | 6/5/2024 | 25 |
| *Karenia brevis* | 1021 | B | 6/5/2024 | 25 |
| *Karenia brevis* | 1021 | C | 6/12/2024 | 26 |
| *Karenia mikimotoi* | 67 | A | 5/29/2024 | 15 |
| *Karenia mikimotoi* | 67 | B | 5/29/2024 | 15 |
| *Karenia mikimotoi* | 67 | C | 6/5/2024 | 15 |
| *Karenia papilionacea* | 1020 | A | 5/29/2024 | 28 |
| *Karenia papilionacea* | 1020 | B | 5/29/2024 | 9 |
| *Karenia papilionacea* | 1020 | C | 6/5/2024 | 25 |
| *Karenia umbella B2* | 1019 | A | 5/29/2024 | 15 |
| *Karenia umbella B2* | 1019 | B | 5/29/2024 | 15 |
| *Karenia umbella B2* | 1019 | C | 6/5/2024 | 15 |

**Table S5.** Metadata of samples used for *Karenia brevis* counts. Cell count data is provided by the Fish and Wildlife Research Institute at <https://geodata.myfwc.com/datasets/myfwc::recent-harmful-algal-bloom-hab-events/>.

| **Collection Date** | **Latitude** | **Longitude** | **Depth (m)** | ***K. brevis* (cells/L)** |
| --- | --- | --- | --- | --- |
| 4/5/2021 | 27.11382 | -82.46883 | 0.5 | 46,500 |
| 4/6/2021 | 26.709683 | -82.261117 | 0.5 | 17,000 |
| 4/29/2021 | 26.93939 | -82.052278 | 0.1 | 11,000 |
| 4/29/2021 | 26.90927 | -82.09481 | 0.1 | 285,311 |
| 6/12/2021 | 28.003935 | -82.792736 | 0.5 | 0 |
| 6/15/2021 | 27.80192 | -82.7746 | 0.5 | 1,298,692 |
| 6/28/2021 | 27.759803 | -82.631267 | 0.5 | 393,333 |
| 7/25/2021 | 27.8009 | -82.8034 | 0.5 | 1,769,218 |
| 7/25/2021 | 27.9006 | -82.8499 | 0.5 | 152,667 |
| 7/25/2021 | 27.687331 | -82.738687 | 0.5 | 390,333 |
| 7/26/2021 | 27.4595 | -82.6982 | 0.5 | 152,667 |
| 11/7/2022 | 27.33375 | -82.57938 | 0.5 | 431,030 |
| 11/14/2022 | 27.33375 | -82.57938 | 2.7 | 76,667 |
| 5/8/2023 | 27.33375 | -82.57938 | 2 | 667 |
| 2/24/2023 | 27.64966 | -83.410366 | 0.5 | 0 |
| 3/27/2023 | 27.64966 | -83.410366 | 0.5 | 0 |
| 2/24/2023 | 27.64966 | -83.2474 | 0.5 | 0 |
| 3/27/2023 | 27.64966 | -83.2474 | 0.5 | 0 |
| 2/24/2023 | 27.64966 | -83.084434 | 0.5 | 1,333 |
| 3/27/2023 | 27.64966 | -83.084434 | 0.5 | 333 |
| 12/5/2022 | 27.64966 | -82.921468 | 0.5 | 4,752,180 |
| 2/24/2023 | 27.64966 | -82.921468 | 0.5 | 12,000 |
| 3/27/2023 | 27.64966 | -82.921468 | 0.5 | 41,333 |
| 2/24/2023 | 27.64966 | -82.84019 | 0.5 | 1,719,010 |
| 2/24/2023 | 27.64966 | -82.84019 | 8.8 | 1,333 |
| 3/27/2023 | 27.64966 | -82.84019 | 0.5 | 94,000 |
| 3/27/2023 | 27.64966 | -82.84019 | 8.8 | 11,333 |
| 4/25/2023 | 27.64966 | -82.84019 | 0.5 | 0 |
| 4/25/2023 | 27.64966 | -82.84019 | 8.7 | 0 |
| 11/17/2022 | 26.33856 | -82.09553 | 0.5 | 576,810 |
| 11/17/2022 | 26.40238 | -82.04633 | 0.5 | 1,462,680 |
| 11/17/2022 | 26.42421 | -82.03075 | 0.5 | 314,167 |
| 11/17/2022 | 26.3897 | -82.14855 | 0.5 | 75,000 |
| 11/17/2022 | 26.500267 | -82.1473 | 0.5 | 24,667 |
| 11/17/2022 | 26.41931 | -82.14855 | 0.5 | 234,125 |
| 11/17/2022 | 26.45062 | -82.16691 | 0.5 | 11,333 |
| 11/17/2022 | 26.41249 | -82.23488 | 0.5 | 392,551 |
| 11/17/2022 | 26.48713 | -82.19907 | 0.5 | 104,055 |
| 11/17/2022 | 26.46784 | -82.23019 | 0.5 | 20,000 |
| 11/17/2022 | 26.54911 | -82.20238 | 0.5 | 283,667 |
| 11/17/2022 | 26.52273 | -82.19497 | 0.5 | 157,284 |
| 11/17/2022 | 26.50999 | -82.19332 | 0.5 | 239,667 |
| 11/17/2022 | 26.49495 | -82.19012 | 0.5 | 196,905 |
| 2/21/2023 | 27.55661 | -82.78781 | 0.5 | 398,000 |
| 2/21/2023 | 27.55661 | -82.78781 | 6 | 315,000 |
| 2/23/2023 | 28.223457 | -82.854093 | 0.9 | 0 |
| 4/25/2023 | 27.5991 | -82.7 | 0.5 | 0 |
| 4/25/2023 | 27.5991 | -82.7 | 5.9 | 0 |
| 4/25/2023 | 27.55661 | -82.78781 | 0.5 | 0 |
| 4/25/2023 | 27.55661 | -82.78781 | 7.2 | 0 |
| 5/4/2023 | 27.957 | -82.622 | 0.5 | 0 |
| 5/9/2023 | 27.33 | -83.359 | 0.5 | 0 |
| 5/9/2023 | 27.489 | -83.417 | 0.5 | 0 |
| 5/12/2023 | 27.8252 | -82.82875 | 0.5 | 0 |

**Table S6.** RT-PCR amplification results for each primer pair on all field-collected samples listed in **Table S1**. Cells with values of 1 denote the presence while those with values of 0 (shaded in grey) denote the absence of RT-PCR products.

| **Sample ID** | **Riboviria1RdRp_F/R** | **Riboviria2 Capsid_F/R** | **Sogarnavirus1RdRp_F/R** | **Sogarnavirus2RdRp_F/R** | **Sogarnavirus3RdRp_F/R** | **Bacillarnavirus1 RdRp_F/R** | **Marnavirus1**  **Capsid_F/R** |
| --- | --- | --- | --- | --- | --- | --- | --- |
| Pool2_12 | 1 | 0 | 0 | 0 | 0 | 0 | 0 |
| Pool2_13 | 1 | 1 | 1 | 0 | 1 | 1 | 0 |
| Pool2_14 | 0 | 0 | 1 | 1 | 1 | 1 | 1 |
| Pool2_15 | 0 | 1 | 1 | 1 | 1 | 0 | 0 |
| Pool2_16 | 1 | 1 | 1 | 1 | 0 | 0 | 0 |
| Pool2_17 | 1 | 1 | 1 | 0 | 1 | 0 | 0 |
| Pool2_18 | 1 | 0 | 1 | 1 | 1 | 0 | 0 |
| Pool1_8 | 1 | 0 | 0 | 1 | 1 | 0 | 0 |
| Pool1_9 | 0 | 0 | 0 | 1 | 0 | 0 | 0 |
| Pool1_10 | 0 | 0 | 1 | 0 | 1 | 0 | 0 |
| Pool1_11 | 0 | 0 | 1 | 0 | 0 | 0 | 0 |
| NP1 | 1 | 0 | 0 | 0 | 1 | 1 | 0 |
| NP2 | 1 | 0 | 0 | 0 | 1 | 1 | 0 |
| NP3 | 0 | 0 | 0 | 0 | 0 | 0 | 0 |
| Sanibel_01 | 1 | 0 | 0 | 0 | 0 | 0 | 1 |
| Sanibel_04 | 1 | 0 | 0 | 0 | 0 | 1 | 0 |
| Sanibel_B14 | 1 | 0 | 0 | 0 | 0 | 1 | 0 |
| Sanibel_D2 | 1 | 0 | 0 | 0 | 0 | 0 | 0 |
| Sanibel_P11 | 1 | 0 | 1 | 1 | 0 | 1 | 0 |
| Sanibel_D14 | 1 | 0 | 0 | 0 | 0 | 1 | 0 |
| Sanibel_E1 | 1 | 0 | 0 | 1 | 0 | 1 | 0 |
| Sanibel_E2A | 1 | 0 | 0 | 0 | 0 | 0 | 0 |
| Sanibel_F1 | 1 | 0 | 0 | 1 | 0 | 1 | 0 |
| Sanibel_F2 | 0 | 0 | 0 | 0 | 0 | 0 | 0 |
| Sanibel_G503 | 1 | 0 | 0 | 1 | 0 | 0 | 0 |
| Sanibel_G506 | 1 | 0 | 0 | 1 | 0 | 1 | 0 |
| Sanibel_G507 | 1 | 0 | 0 | 1 | 0 | 1 | 0 |
| Sanibel_G508 | 1 | 0 | 0 | 1 | 0 | 1 | 0 |
| STING_1_1m | 0 | 0 | 1 | 1 | 0 | 0 | 0 |
| STING_1_8m | 0 | 0 | 0 | 0 | 1 | 0 | 0 |
| STING_13_2m | 0 | 0 | 0 | 0 | 1 | 0 | 0 |
| ROME04_120522_surface | 0 | 0 | 0 | 0 | 1 | 0 | 0 |
| ROME01_022423_surface | 0 | 0 | 0 | 0 | 0 | 0 | 0 |
| ROME02_022423_surface | 0 | 0 | 0 | 0 | 0 | 0 | 0 |
| ROME03_022423_surface | 0 | 0 | 0 | 1 | 1 | 0 | 0 |
| ROME04_022423_surface | 0 | 0 | 0 | 0 | 0 | 0 | 0 |
| ROME05_022423_surface | 0 | 0 | 0 | 0 | 0 | 0 | 0 |
| ROME05_022423_bottom | 0 | 0 | 1 | 0 | 0 | 0 | 0 |
| ROME01_032723_surface | 0 | 0 | 0 | 0 | 0 | 0 | 0 |
| ROME02_032723_surface | 0 | 0 | 0 | 0 | 0 | 0 | 0 |
| ROME03_032723_surface | 0 | 0 | 0 | 0 | 0 | 0 | 0 |
| ROME04_032723_surface | 0 | 0 | 0 | 1 | 1 | 0 | 0 |
| ROME05_032723_surface | 0 | 0 | 1 | 1 | 1 | 0 | 0 |
| ROME05_032723_bottom | 0 | 0 | 0 | 1 | 1 | 0 | 0 |
| ROME05_042523_surface | 0 | 0 | 0 | 0 | 0 | 0 | 0 |
| ROME05_042523_bottom | 0 | 0 | 0 | 1 | 1 | 0 | 0 |
| E01 Surface | 1 | 0 | 0 | 1 | 1 | 0 | 0 |
| E01 Bottom | 0 | 0 | 0 | 1 | 0 | 0 | 0 |
| CS00A Surface | 0 | 0 | 1 | 1 | 0 | 0 | 0 |
| CS00A Bottom | 0 | 0 | 0 | 1 | 0 | 0 | 0 |
| GOMETS2 | 0 | 0 | 0 | 0 | 1 | 0 | 0 |
| GOMETS11 | 0 | 0 | 1 | 0 | 0 | 0 | 0 |
| GOMETS13 | 0 | 0 | 0 | 0 | 1 | 0 | 0 |
| GOMETS17 | 0 | 0 | 0 | 1 | 1 | 0 | 0 |
